# CircSLC22A3 inhibits the invasion and metastasis of ESCC via the miR-19b-3p/TRAK2 axis and by reducing the stability of m^6^A-modified ACSBG1 mRNA

**DOI:** 10.1186/s12885-025-14390-8

**Published:** 2025-05-30

**Authors:** Yingjie Pan, Hang Yang, Jiayi Zhang, Ruolan Zhang, Yun Liu, Jun Bie, Qiaoling Chen, Yan Qiao, Kang Liu, Guiqin Song

**Affiliations:** 1 Institute of Tissue Engineering and Stem Cell Research, The Second Clinical College of North Sichuan Medical College, Beijing Anzhen Nanchong Hospital of Capital Medical University & Nanchong Central Hospital, Nanchong, 637000 P.R. China; 2 Institute of Basic Medicine and Forensic Medicine, North Sichuan Medical College, Nanchong, 637100 P.R. China

**Keywords:** ESCC, circSLC22A3, miR-19b-3p, *TRAK2*, IGF2BP1, *ACSBG1*

## Abstract

**Background:**

Esophageal squamous cell carcinoma (ESCC) is a major contributor to cancer-related deaths, driven by its invasive and metastatic nature. Circular RNAs (circRNAs) are increasingly recognized as regulators of cancer progression, primarily through miRNA sponging and interactions with RNA-binding proteins. Their dysregulation has been linked to the development of in various cancers. The present study aimed to investigate the potential involvement of circSLC22A3 in the pathogenesis of ESCC.

**Methods:**

CircSLC22A3 expression in ESCC tissues and cells was analyzed using transcriptome sequencing and RT-qPCR. Its circular structure was validated through Sanger sequencing, agarose gel electrophoresis, RNase R digestion, and random priming assays. Subcellular localization was determined by nucleoplasmic separation and fluorescence in situ hybridization (FISH). Clinical correlations were assessed via tissue microarrays. Functional roles of circSLC22A3 in ESCC progression were investigated through in vitro and in vivo assays. Downstream miR-19b-3p and target gene TRAK2 were screened by bioinformatics analysis and RT-qPCR, with binding confirmed via luciferase reporter assays. RNA pulldown combined with RNA immunoprecipitation (RIP) identified IGF2BP1 as a circSLC22A3-interacting protein. RNA-seq and RT-qPCR revealed ACSBG1 as a key downstream effector. IGF2BP1-mediated m^6^A modification of ACSBG1 was mapped by MeRIP-seq and RIP, with mRNA stability assessed via Actinomycin D assay. ACSBG1 expression and biological function in ESCC were confirmed by immunohistochemistry, RT-qPCR, and functional assays.

**Results:**

Significant downregulation of circSLC22A3 was observed in both ESCC tissues and cell lines. Overexpression of circSLC22A3 significantly reduced ESCC cells’ migration and invasion capabilities. Mechanistic investigation revealed that circSLC22A3 played a pivotal role in the invasion and metastasis of esophageal cancer through distinct pathways. On one hand, circSLC22A3 functioned as a miR-19b-3p sponge to augment trafficking kinesin protein 2 (TRAK2) expression, while, on the other hand, circSLC22A3 formed a protein-RNA complex with IGF2BP1, resulting in the degradation of acyl-CoA synthetase bubblegum family member 1 (ACSBG1) mRNA through the recognition of m^6^A modification, thereby suppressing invasion and metastasis of ESCC.

**Conclusions:**

The present study identified circSLC22A3 as a new tumor suppressor that inhibited ESCC progression through both the circSLC22A3/ miR-19b-3p/ *TRAK2* and circSLC22A3/ IGF2BP1/ *ACSBG1* axes.

**Supplementary Information:**

The online version contains supplementary material available at 10.1186/s12885-025-14390-8.

## Introduction

Esophageal squamous cell carcinoma (ESCC), the dominant subtype of esophageal cancer, is one of the most common digestive tumors worldwide [1]. Despite advances in treatment, the 5-year overall survival for ESCC remains < 30% [2, 3]. Relapse and metastasis are the main causes of the unfavorable outcome in ESCC. In addition, one of the main reasons for treatment failure and poor prognosis in patients with ESCC is that the total recurrence rate after radical surgery is as high as 27-52.4%, while the local recurrence rate is as high as 32.6–49%, and the distant metastasis rate in patients with positive lymph nodes is as high as 19.8–61.3% [4–6]. Consequently, further investigation into the molecular mechanisms affecting the metastatic spread of ESCC is urgent to obtain more effective antitumor treatment strategies.

Circular RNAs (CircRNAs) are a class of closed-loop structural molecules consisting of linear RNA precursors that are reverse spliced to form noncoding RNA that are highly stable and exhibit cell- and tissue-specific expression patterns [7–9]. Previous studies have shown that dysregulated circRNAs are prevalent in tumors and are key regulators involved in tumorigenesis, playing important roles in the malignant proliferation, apoptosis, metastasis and drug resistance of tumor cells [8, 10–15].

In recent years, there has been an increase in research conducted on the role of circRNAs in ESCC [16–22]. There is growing evidence that circRNAs act as microRNA (miRNA or miR) sponges to intervene in the regulation of ESCC metastasis and invasion. For example, upregulation of circCNOT6L in ESCC has been observed, which can enhance the proliferation, migration, and invasion of ESCC through the miR4270/*MCM3* axis [23]. Additional studies showed that circ-TNRC6B interacted with miR-452-5p, thereby attenuating its facilitative impact on the migratory and invasive potential of ESCC [24]. Additionally, circRNAs can interact with RNA-binding proteins (RBPs), and function as a protein sponge, bait, or scaffold molecule, thereby influencing the cellular localization of RBPs and modulating the stability of target mRNA molecules [25, 26]. For instance, circNEIL3 inhibits colorectal cancer metastasis by promoting the proteasomal degradation of YBX1 [27]. Hsa_circ_0004296 can inhibit prostate cancer metastasis by affecting ETS1 mRNA through interaction with EIF4A3 [28]. Hsa_circ_0006646 is a metastasis-associated circRNA upregulated in hepatocellular carcinoma cells and tissues that prevents the interaction between nucleolin and the E3 ligase TRIM21, thereby reducing the ubiquitinated degradation of nucleolin [29].

The regulatory function of m^6^A is mainly performed by three homologous factors, which are called ‘writer’, ‘eraser’ and ‘reader’ [30]. A large number of m^6^A RNA readers, including proteins containing the YT521-B homology domain (YTH) (YTHDF1, YTHDF2, YTHDF3, YTHDC1 and YTHDC2) and insulin-like growth factor 2 mRNA-binding proteins (IGF2BPs) (IGF2BP1, IGF2BP2 and IGF2BP3) recognize m^6^A-modified RNAs and control mRNA fate by affecting its stability, translation, alternative splicing, and subcellular localization [31–33]. Among them, IGF2BP1 can regulate gene stability by recognizing m^6^A-modifying RNAs of target genes, thus further promoting cancer progression [31, 34]. For instance, in non-small cell lung cancer, circNDUFB2 acted as a scaffold to enhance the interaction between TRIM25 and IGF2BPs, and the resulting ternary complex formed by TRIM25, circNDUFB2 and IGF2BPs promoted the ubiquitination and degradation of IGF2BPs while also stimulating immune response mechanisms that inhibited tumor growth and metastasis [35]. In bladder cancer, circCPTPRA interacted with IGF2BP1 to impact the stability of its target mRNA molecules [36].

CircRNAs are currently in their nascent stage and their functional mechanism remains unclear, despite its utilization in ESCC research. The present study identified a notable decrease in the expression of cirRNAs in ESCC. Specifically, circSLC22A3, also known ashsa_circ_0078607, a circRNA derived from solute carrier family 22, member 3 (SLC22A3) pre-mRNA, was identified as a subject of investigation. Through scratch migration, Transwell, and RNA stability assays, as well as mouse experiments utilizing tail vein and nude mouse xenograft models, it was determined that circSLC22A3 exerted a negative influence on the metastatic potential of ESCC. The molecular network mechanism of circSLC22A3 in ESCC was further investigated. Specifically, circSLC22A3 competitively bound to miR-19b-3p, thereby attenuating its inhibitory effect on Trafficking Kinesin Protein 2 (*TRAK2*) expression and suppressing epithelial-mesenchymal transition progression in ESCC cells, ultimately impeding metastasis. Additionally, circSLC22A3 formed a protein-RNA complex with IGF2BP1 and facilitated degradation of acyl-CoA Synthetase Bubblegum Family Member 1 (*ACSBG1*) mRNA through recognition of m^6^A modification, resulting in inhibition of migration and invasion in ESCC. Collectively, the present findings shed light on the molecular mechanisms by which circSLC22A3 affects the progression of ESCC by inhibiting its migration and invasion, and offer a foundation for future investigations into novel biomarkers and therapeutic targets for ESCC.

## Materials and methods

### Tissue specimens

Clinical tissue specimens: ESCC and corresponding adjacent tissue were obtained form 6 cases of ESCC. The tissues were removed during thoracic surgery at Nanchong Central Hospital between November 2018 and March 2019, collected from ESCC patients aged 48–75, and were used for transcriptome sequencing. (GSE263647). None of the patients had received any treatment prior to the surgery. Signed informed consent was obtained from all patients. The study protocol was approved by the ethics committee of Nanchong Central Hospital (approval no.2019095).

Tissue microarray: Human-derived ESCC tissue microarray was derived from the National Human Genetic Resource Sharing Service Platform (platform no. 2005 DKA 21300; approval no. SHYJS-CP-2004003). The data from tissue microarray are presented in Tables SI and II).

Immunohistochemical analysis: Sections were obtained from 125 patients with ESCC aged 47–81 who were subjected to tumor resection at Nanchong Central Hospital from January 2019 to December 2022. The selected patients did not receive chemoradiotherapy before and after the surgery. The pathological data of ESCC patients involved in the immunohistochemistry experiment are approval by Ethics Committee of North Sichuan Medical College [ (2021) no. 42], detail information are presented in Table SIII-V.

### Bioinformatics database network

The following databases were employed in the present study: The Cancer Genome Atlas (TCGA) (https://www.cancer.gov/ccg/research/genome-sequencing/tcga); miRanda (http://www.microrna.org/microrna); CircInteractome Database (https://circinteractome.nia.nih. gov); starBase (https://starbase.sysu.edu. cn); TargetScan (www.targetscan. org); miRWalk (http://mirwalk.umm. uni-heidelberg. de); RBPsuite (http://www.csbio.sjtu.edu.cn/bioinf/RBPsuite/); and The Human Protein Atlas (https://www.proteinatlas.org/).

### Cell culture

The human ESCC cell lines KYSE150 and TE-1 were purchased from Shanghai Chinese Academy of Sciences Cell Bank (Shanghai, China), KYSE30 and KYSE410 were purchased from Hunan Fenghui Biotechnology Co., Ltd. (Hunan, China). KYSE510 was purchased from Wuhan Shann Biotechnology Co. Ltd. (Wuhan, China). The ESCC cells lines were cultured in RPMI-1640 medium (Gibco; Thermo Fisher Scientific, Inc.) with 10% fetal bovine serum (Bovogen Biologicals, Pty Ltd.). HET-1A cells were cultured in Bronchial Epithelial Cell Growth Medium (Gibco; Thermo Fisher Scientific, Inc.). Cells were incubated at 37 ˚C in 5% CO_2_. All cell lines were free of mycoplasma contamination and verified by STR analysis.

The lentiviral overexpression plasmid of circSLC22A3 was synthesized by Shanghai Jikai Gene Chemical Technology Co., Ltd. *TRAK2*/*IGF2BP1* overexpression plasmids, miR-19b-3p mimic and inhibitor, *IGF2BP1*/*ACSBG1* small interfering RNAs (siRNAs) and short hairpin RNAs (shRNAs) were purchased from Shanghai GenePharma Co., Ltd. Transfection was performed using Lipofectamine™ 2000, Lipofectamine™ 3000 and Lipofectamine™ RNAiMAX (Invitrogen; Thermo Fisher Scientific, Inc.) according to the manufacturer’s instructions. The sequences of the overexpression plasmids, miRNA mimic and inhibitor, siRNAs and shRNAs are listed in Table SVI.

### RNase R treatment

Total RNA (2 µg/group) from KYSE30 cells was treated with RNase R (Thermo Fisher Scientific, Inc.) and incubated at 37 ˚C for 30 min, followed by heat inactivation at 70 ˚C for 10 min. Equal volumes of RNA were then reverse transcribed, and the expression levels of circRNA and its parental gene mRNA were detected by RT-qPCR.

### Nuclear-cytoplasmic fractionation

Cytoplasmic and nuclear RNA were separated using Nuclear and Cytoplasmic Extraction Reagents (Thermo Fisher Scientific, Inc.) following the manufacturer’s instructions. GAPDH and U6 were used as reference genes in the cytoplasm and nucleus, respectively.

### RNA fluorescence in situ hybridization (FISH)

The expression of circSLC22A3 in human ESCC tissue chips, and the cellular localization of circSLC22A3 and miR-19b-3p were investigated using FISH. Human ESCC tissue microarray was provided by Shanghai Outdo Biotech Co., Ltd., while Shanghai GenePharma Co., Ltd. developed and synthesized the Cy3-labeled circSLC22A3 and miR-19b-3p probe. Images were acquired using a fluorescent confocal microscope. The sequences for circSLC22A3 and miR-19b-3p probe are listed in Table SVII.

### Reverse transcription quantitative PCR (RT-qPCR)

Total RNA was extracted from each group using FastPure Cell/Tissue Total RNA Isolation Kit (Vazyme Biotech Co., Ltd.). The RNA concentration was measured using a Nano Drop ONE spectrophotometer (Thermo Fisher Scientific, Inc.). Next, the RNA was reverse-transcribed to cDNA with HiScript^®^ III RT SuperMix (Vazyme Biotech Co., Ltd.). RT-qPCR has the following thermocycling conditions: 95 ˚C for 3 min, followed by 95 ˚C for 10 s and 60 ˚C for 30 s, for a total of 40 cycles. Relative expression of the target gene was calculated by the 2^−ΔΔCq^ method [37], using GAPDH or U6 as the internal reference gene for normalization. The sequences of the primers used in the present study are listed in Table SVIII.

### Dual-luciferase reporter assay

Synthetic plasmids and their corresponding sequences were designed by Shanghai GenePharma Co., Ltd. Luciferase reporter plasmids and RNA were transfected in cells at 60-80% confluency according to the manufacturer’s instructions of Lipofectamine™ 2000 (Thermo Fisher Scientific, Inc.). After 48 h, dual luciferase assay was performed with Dual-Luciferase^®^ Reporter Assay System (Promega Corporation).

### Transwell assay

Transwell Boyden chambers (BD Biosciences) with 8 μm pore polycarbonate filters were used for in vitro cell migration assays. A total of 600 µl 20% serum-containing medium was added to the Transwell lower chamber, while 200 µl serum-free medium containing 3 × 10^5^ cells was added to the upper chamber. For the invasion assay, Matrigel was added to the upper chamber, and 150 µl serum-free medium containing 1 × 10^6^ cells was added. After 24 h incubation at 37 ˚C in the presence of 5% CO_2_, the cells were fixed with 20% methanol for 30 min and then stained with 0.1% crystal violet solution. Next, the cells in the Transwell chamber were gently swabbed, photographed and counted. The experiments were performed in a triplicate.

### Western blotting

TE-1 and KYSE30 cells were harvested after transfection and then total protein was extracted after cell lysis with RIPA lysis buffer (Epizyme; Ipsen Pharma). The concentration of total protein was determined using a BCA protein assay kit (Beyotime Institute of Biotechnology). Protein samples were separated by 10% SDS-PAGE and transferred to a nitrocellulose membrane. After blocking with 5% skim milk, primary antibody were added for overnight incubation at 4˚C. Next, HRP-labeled secondary antibodies (Proteintech; cat. no. SA00001-2) were added, and incubated at room temperature for 1 h. Subsequently, the membranes were washed with PBS-Tween 20 and subjected to enhanced chemiluminescence (Vazyme Biotech Co., Ltd.; cat. no. E423-01) exposure in a dark chamber for development and visualization of the protein bands. The gray values of the protein bands were analyzed with Image J software (Version 1.53). The primary antibodies used in the present study were against E-cadherin (cat. no. ET1607-75; 1:1,000), Snail (cat. no. 13099-1-AP; 1:1,000), vimentin (cat. no. ET1610-39; 1:500), TRAK2 (cat. no. 13770-1-AP; 1:500) and IGF2BP1 (cat. no. FNab04171; 1:1,000). All the above antibodies were purchased from Wuhan Sanying Biotechnology Co., Ltd. (Wuhan, China).

### Immunohistochemistry

The expression and distribution of TRAK2, IGF2BP1 and ACSBG1 were detected by immunohistochemical staining in the tissues of patients with ESCC and in their corresponding para-cancer tissues. The slices were incubated in an oven at 65 ˚C for 4 h, followed by gradient dewaxing and hydration. Next, the slices were heated and subjected to antigen repair in citrate buffer, followed by blocking of the endogenous catalase activity with 3% H_2_O_2_ according to the instructions of the generic immunohistochemistry two-step test kit of Beijing Zhongshan Golden Bridge Biotechnology Co., Ltd. (Beijing, China) (cat. no. PV-9000). The following antibodies were used in this experiment. Anti TRAK2 (1:50), anti-IGF2BP1 (1:50), and anti-ACSBG1 (cat. no. 160771-1-AP; 1:100) antibodies.

### Wound healing test

TE-1 and KYSE30 cells at logarithmic-phase proliferation of NC, overexoression and siRNA groups were seeded in 6-well plates, and streaked when the cells confluence reached ~ 70%, Next, the cells werewashed three times with PBS, and 2 ml serum-free medium was added. Photographs were obtained at 0 h and 24 h to calculate the cell scratch healing rate. The experiments were performed in a triplicate.

### Flow cytometry

Each group of transfected cells was digested with EDTA-free trypsin, collected, washed twice with precooled PBS. 100 µl of 1 × binding buffer was added, and the cells were gently blown until a single-cell suspension was formed. 5 µl of annexin V-FITC and 5 µl of propidium iodide solution (Vazyme Biotech Co., Ltd.) were added, mixed well, and treated at room temperature in the dark for 15 min before being detected by flow cytometry.

### RNA pull-down assay

Biotin-labeled circSLC22A3 and oligonucleotide probes, which were designed and synthesized by Shanghai GenePharma Co., Ltd, were mixed with streptavidin magnetic beads in RIPA buffer for 4 h. Then, the KYSE30 cell lysate was incubated with the probes complex for 12 h at 4 ˚C. After purification, RT-qPCR was conducted to detect the enriched circSLC22A3 and miRNAs, while silver staining was employed for protein enrichment detection.

### RNA Immunoprecipitation (RIP) assay

RIP assay was conducted with a RIP kit (Millipore, USA) according to the manufacturer’s instructions. Coprecipitated RNAs were subsequently identified through RT-qPCR utilizing specific primers.

### Methylated RIP sequencing (MeRIP-seq)

Total RNA was isolated from cells and subjected to polyadenylated RNA enrichment using VAHTS mRNA Capture Beads(VAHTS, cat. no. N401-01/02). A specific anti-m^6^A antibody (1:100) (cat. no. 202 203; Synaptic Systems was utilized for m^6^A IP at 4 ˚C. Subsequently, the enriched RNA fragments were subjected to high-throughput sequencing.

### mRNA stability test

mRNA stability was assessed by conducting an RNA decay experiment using actinomycin D (Act D) to validate its degradation kinetics. After treating cells with Act D, total RNA was extracted at 0 h, 1 h, 2 h and 3 h, and mRNA expression was detected by RT-qPCR.

### In vivo tumor model

Eight male BALB/c nude mice (5 weeks old) were ordered from Beijing Huafukang Biotechnology Co., Ltd. (Beijing, China) for the establishment of a lung metastasis model. They were divided into two groups, with 4 mice in each group. The body weight of the mice was about 20 g. They were raised in the Animal Center of North Sichuan Medical College (Nanchong, China). The mice were maintained at room temperature (22 ± 1˚C) with a 12/12 h light/dark cycle and ad libitum access to food and water. Each group received a tail vein injection with fluorescent vectors stably overexpression circSLC22A3 or vector KYSE30 cells at a concentration of 2 × 10^6^ cells. At 8 weeks post-injection, the mice were euthanized, and the fluorescence images of xenografts were acquired using an in vivo imaging system. Animals were checked daily, and any animal found unexpectedly to be moribund, cachectic or unable to obtain food or water were euthanized, and the mice were intravenously injected with 150 mg/kg pentobarbital sodium for euthanasia. None of the mice reached the humane endpoints in this study.

Xenograft tumor models were established by subcutaneously injecting 150 µl PBS containing 1.2 × 10^6^ sh-ACSBG1 KYSE30 cells or sh-NC KYSE30 cells into mice. Every 3 days, the body weight and size of subcutaneous tumors were evaluated. After 4 weeks, before the tumor volume exceeded 1,000 mm³, the mice were intravenously injected with 150 mg/kg pentobarbital sodium for euthanasia. Then a pulse examination will be carried out after cardiac arrest to confirm death. The animal experiments were approved by Ethics Committee of North Sichuan Medical College (Nanchong, China) (approval no. 202144).

### Statistical analysis

The experimental data were analyzed using the statistical software GraphPad Prism 8.0 (GraphPad; Dotmatics). Measurement data that followed a normal distribution were represented asthe mean ± standard deviation. Experiments were repeated independently repeated three times. Comparisons between two groups were conducted using an independent sample Student’s t-test, paired groups were tested using paired t-test, while comparisons between multiple groups were performed using analysis of one-way ANOVA. Survival curves were generated using the Kaplan-Meier method and assessed using the log-rank test. *P* < 0.05 was considered to indicate a statistically significant difference.

## Results

### Characteristics of circSLC22A3 in ESCC

**Fig. 1:**
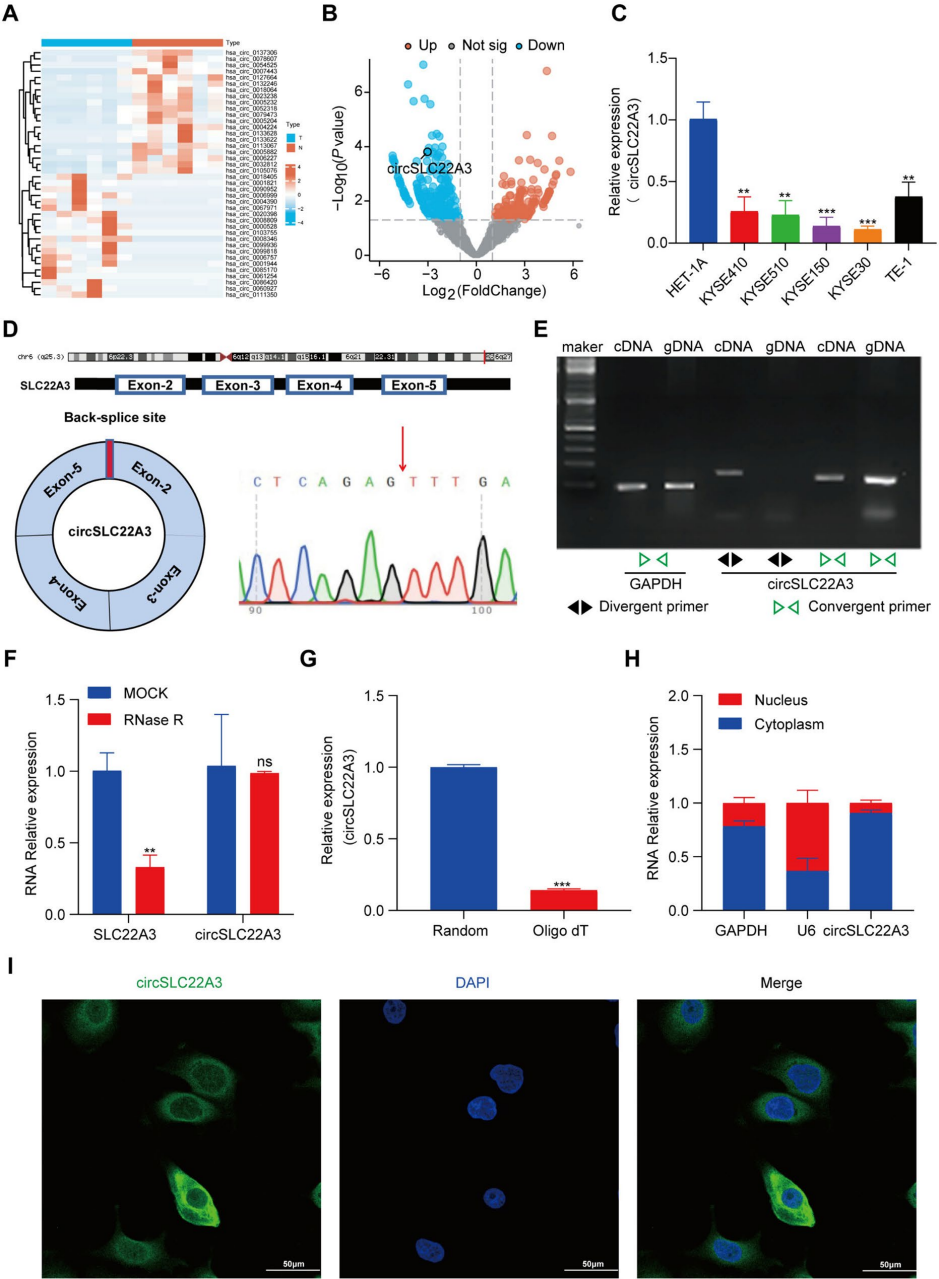
The characterization of circSLC22A3 in ESCC: (**A**) Cluster Analysis Heatmap. (**B**) Difference Analysis Volcano Plot. (**C**) CircSLC22A3 expression in ESCC cells lines and the human normal esophageal epithelial cells. (**D**) Splicing site of circSLC22A3 was validated by Sanger sequencing. (**E**) PCR assay with divergent and convergent primers showing the amplification of circSLC22A3 from cDNA or gDNA of ESCC cell. (**F**) RNase R digestion assay showed that circSLC22A3 was resistant to RNase R. (**G**) Compared with reverse transcription using Oligo(dT) primer, the expression of circSLC22A3 was significantly increased after reverse transcription using random primer. (**H**) RT-qPCR was used to detect the distribution of circSLC22A3 in the nucleus and cytoplasm of ESCC. (**I**) RNA FISH detecting circSLC22A3’s subcellular localization. Scale bar, 50 μm. *, *P* < 0.05. **, *P* < 0.01. ***, *P* < 0.001

The present study aimed to conduct comprehensive transcriptome sequencing on 6 pairs of postoperative cancerous and adjacent tissues obtained from patients diagnosed with ESCC, in order to identify aberrant expression patterns of circRNA. The findings indicated the presence of 291 differentially expressed circRNAs in ESCC, comprising 122 circRNAs with increased abundance and 169 circRNAs with reduced abundance (|log_2_fold-change|≥2, *P* < 0.05). The heatmap visually demonstrated distinct grouping and clustering patterns (Fig. 1A). The volcano plots illustrated the variation in circRNA expression between cancerous and normal samples (Fig. 1B). The primary objective of the present study was to examine circRNAs that displayed notable down-regulation in ESCC. The verification of the sequencing outcomes was accomplished by RT-qPCR. The findings obtained indicated a down-regulation of circSLC22A3 in ESCC cells in comparison to normal esophageal epithelial cells (Fig. 1C).

It is worth noting that circSLC22A3 originates from exons 2–5 of the *SLC22A3* gene. To confirm the authenticity of circSLC22A3 as a circRNA, Sanger sequencing was employed to verify the presence of the circSLC22A3 back-splicing junction (Fig. 1D). Additionally, RT-qPCR with agarose electrophoresis was performed, which revealed the amplification of circSLC22A3 using divergent primers in both cDNA and genomic samples. However, it should be noted that convergent primers could only be successfully amplified in cDNA (Fig. 1E). Furthermore, the RNase R digestion assay demonstrated that circSLC22A3 exhibited resistance to RNase R, while linear SLC22A3 was degraded upon treatment with RNase R (Fig. 1F). Notably, when comparing the results of RT using an Oligo(dT) primer with those of RT using a random primer, it was observed that the expression of circSLC22A3 was significantly increased after RT using a random primer, suggesting the absence of a poly(A) tail in circSLC22A3 (Fig. 1G). Given the relevance of circRNA localization to its in vivo biological function at the molecular level, cytoplasmic and nuclear RNA were separately extracted for subsequent RT-qPCR analysis. By utilizing GAPDH as the cytoplasmic reference and U6 as the nuclear reference, the present findings indicated that circSLC22A3 was localized in both the nucleus and cytoplasm, albeit it was predominantly localized in the cytoplasm (Fig. 1H). The subcellular localization of circSLC22A3 was further confirmed through FISH analysis to be predominant in the cytoplasm (Fig. 1I).

### Clinical relevance of circSLC22A3 in ESCC

**Fig. 2:**
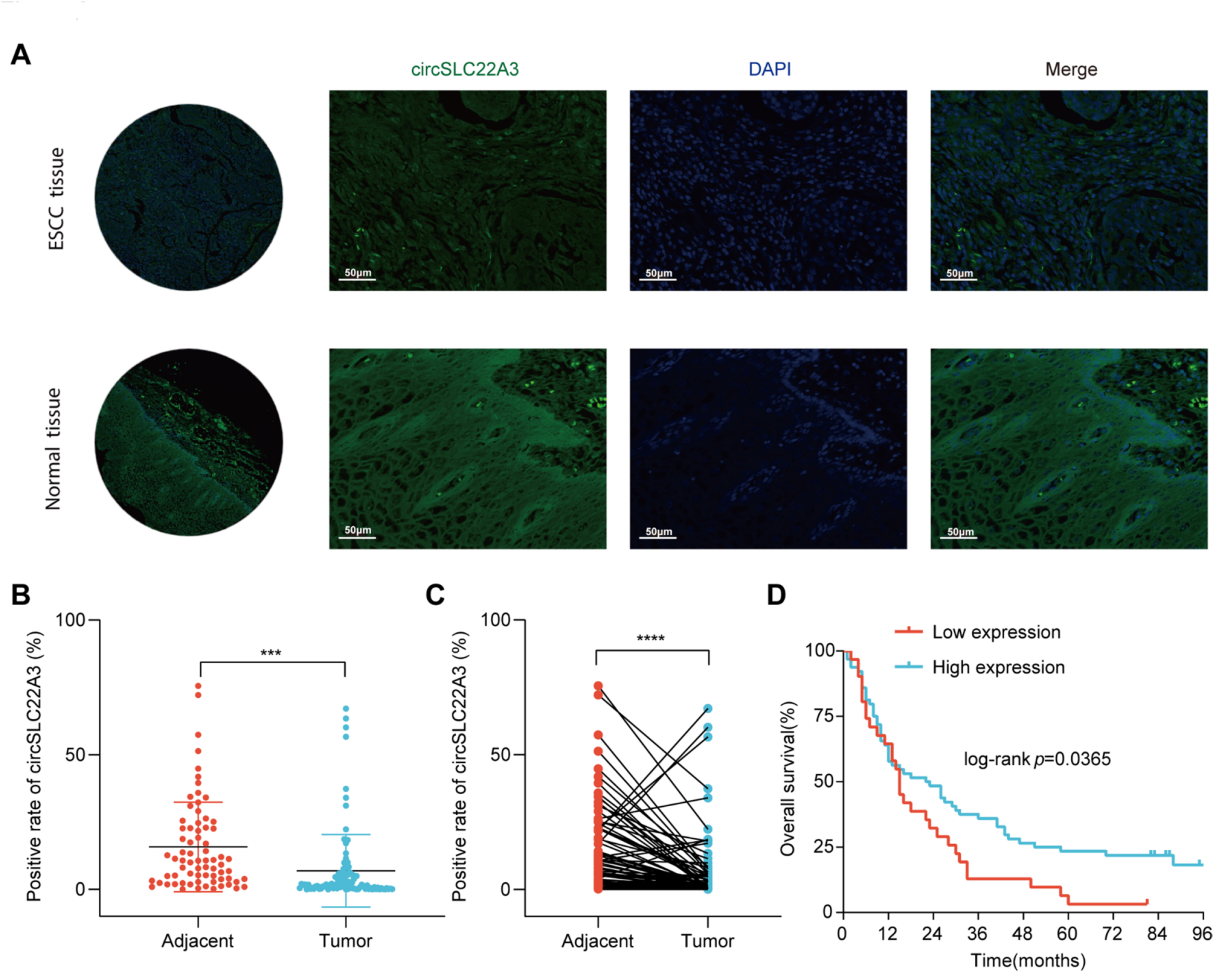
Clinical significance of circSLC22A3 in ESCC: (**A**) Tissue microarray-based RNA FISH analysis showed that circSLC22A3 was lowly expressed in ESCC tissues compared to corresponding adjacent tissues. (**B**) In unpaired 97 ESCC tissues and 72 normal tissues circSLC22A3 expression abundance was significantly lower in ESCC tissues compared to normal tissues. (**C**) In paired 71 ESCC tissues, circSLC22A3 expression abundance was significantly lower in ESCC tissues compared to normal tissues (**D**) Kaplan–Meier analysis showed that patients with circSLC22A3 low expression had a shorter overall survival. Scale bar, 50 μm. ***, *P* < 0.001. ****, *P* < 0.0001

To further explore the clinical significance of circSLC22A3, the expression level of circSLC22A3 was detected by tissue microarray (Fig. 2A). It was found that the expression of circSLC22A3 was significantly reduced in ESCC tissues compared with corresponding adjacent tissues (Fig. 2B and C). Furthermore, the association between circSLC22A3 expression and prognosis in patients with ESCC was analyzed by Kaplan–Meier analysis. Patients with low expression of circSLC22A3 had a shorter overall survival (Fig. 2D). Furthermore, circSLC22A3 low expression was an independent prognostic factor of ESCC.

### CircSLC22A3 attenuates ESCC cell invasion and migration in vitro and in vivo

**Fig. 3:**
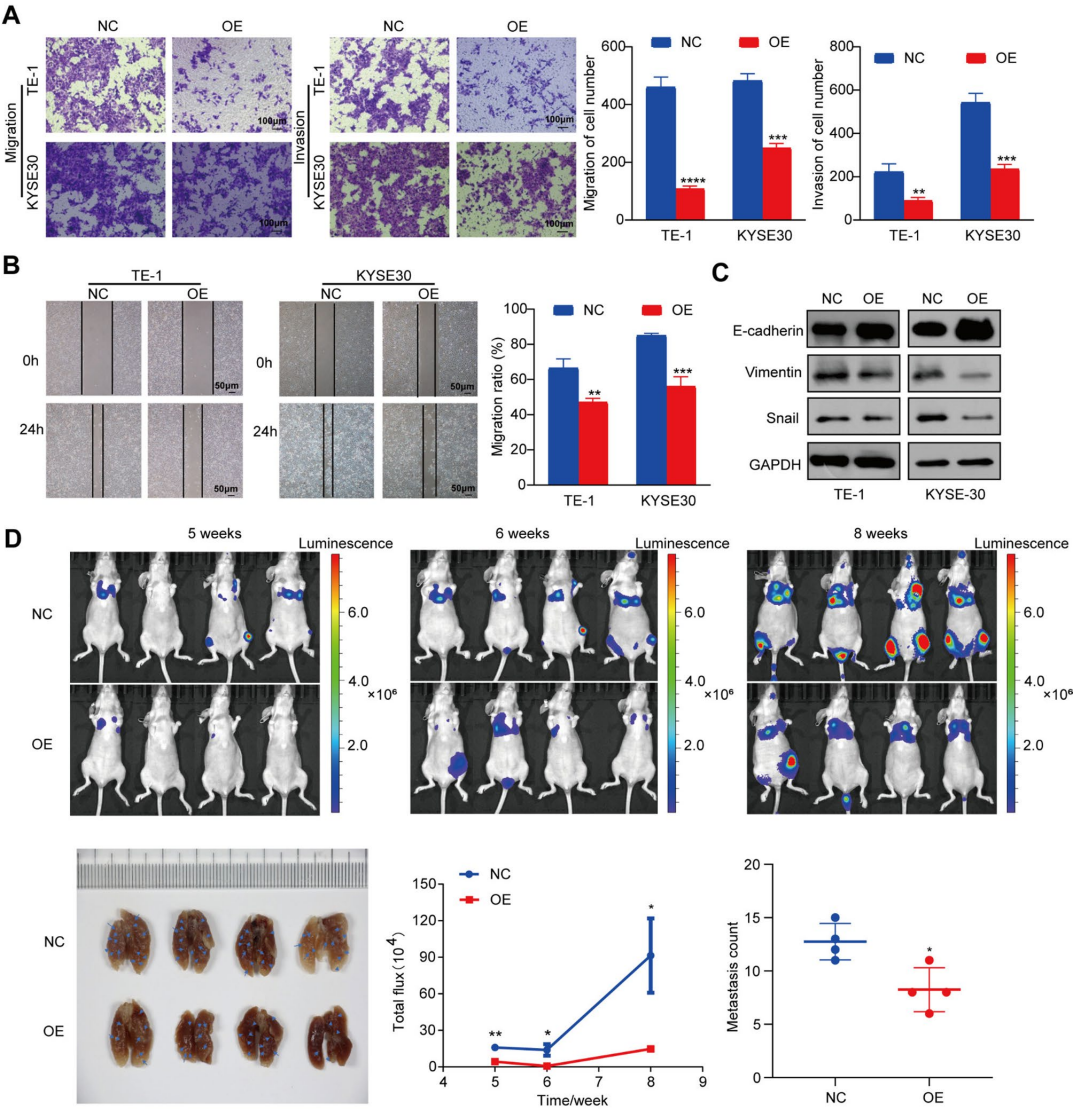
CircSLC22A3 attenuated ESCC cell invasion and migration both in vitro and in vivo: (**A**) Transwell assay results showed that overexpression of circSLC22A3 resulted in a significant reduction in the number of migrating and invasive cells. (**B**) Overexpression of the circSLC22A3 greatly inhibited the migratory capacity of TE-1 and KYSE30 cells in the Scratch wound healing experiments. (**C**) Western blot showed changes in tumor metastasis-related proteins. (**D**) The luciferase images and the gross observation of lung metastases in mice injected with KYSE30/control and KYSE30/circSLC22A3 cells (*n* = 8). Scale bar, 50 μm and 100 μm. *, *P* < 0.05. **, *P* < 0.01. ***, *P* < 0.001. ****, *P* < 0.0001

To investigate the biological role of circSLC22A3 in ESCC cells, lentivirus infection was employed to establish TE-1 and KYSE30 cell lines with stable overexpression of circSLC22A3. The effectiveness of infection was confirmed by RT-qPCR, which demonstrated that overexpression of circSLC22A3 was induced by the lentiviral vector (Fig. S1A). The results of Cell Counting Kit-8 assay CCK-8 experiment and flow cytometry analysis indicated that the overexpression of circSLC22A3 did not have a significant impact on the proliferation of ESCC cells (Fig. S1B and S1C). Subsequently, the impact of circSLC22A3 gene overexpression on the invasive and migratory capabilities of ESCC cells was assessed. Transwell and wound healing assay showed that the migration and invasion abilities of TE-1 and KYSE30 cells were significantly impaired after overexpression of circSLC22A3 (Fig. 3A and B). To assess the impact of circSLC22A3 overexpression on metastasis-related proteins, namely E-cadherin, vimentin and Snail, western blot analysis was conducted. The findings indicated that overexpression of circSLC22A3 led to a significant increase in the expression of E-cadherin protein in TE-1 and KYSE30 cells, while the relative expression of vimentin and Snail proteins was significantly decreased (Fig. 3C). These results collectively supported the notion that circSLC22A3 played a role in inhibiting the progression of ESCC by suppressing the invasion and migration of ESCC cells. To further elucidate the biological functions of circSLC22A3 in vivo, a mouse model of lung metastasis was established. KYSE30 cells with stably expressing control vector or overexpressing circSLC22A3 were intravenously injected into the tail vein of BALB/c nude mice. Metastatic tumor nodules were observed at 5 weeks, 6 weeks and 8 weeks by using an animal imaging system. When the mice were dissected after 8 weeks, those injected with circSLC22A3 overexpressing cellsfewer metastatic nodules in the lungs than mice injected with control cells (Fig. 3D).

### CircSLC22A3 function as a sponge for miR‑19b‑3p

**Fig. 4:**
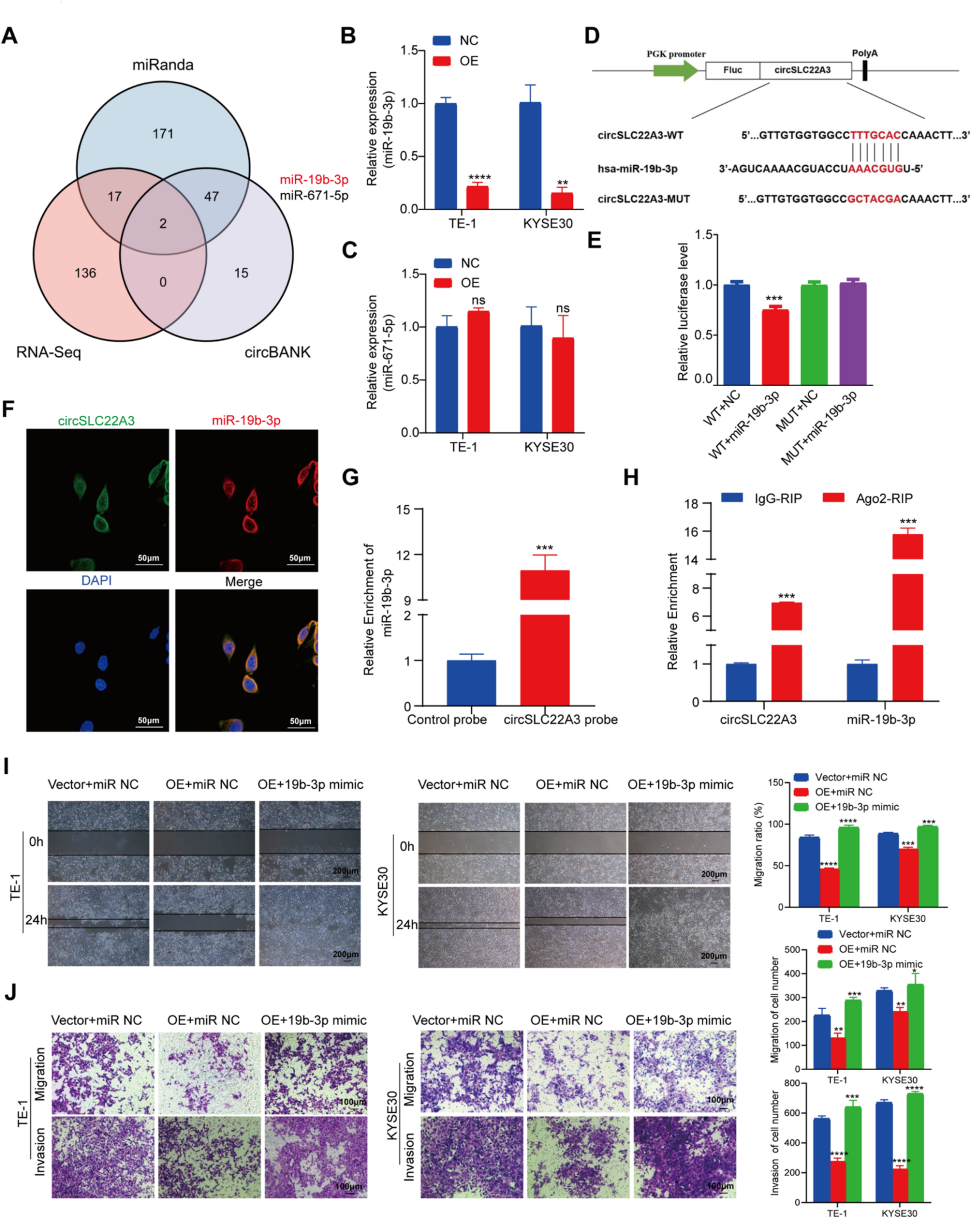
CircSLC22A3 function as a sponge for miR‑19b‑3p: (**A**) Venn diagram showing the predicted miRNAs that circSLC22A3 might interact. (**B, C**) The RT-qPCR showing the mRNA level change of the predicated targets after over expression of circSLC22A3. (**D**) A schema of circSLC22A3 wild-type (WT) and mutant (Mut) luciferase reporter vectors. (**E**) Dual-luciferase reporter gene findings demonstrated that miR-19b-3p mimic inhibited the luciferase activity of circSLC22A3-WT. (**F**) The images captured by confocal microscopy indicate that circSLC22A3 is co-localized with miR-19b-3p in the cytoplasm. (**G**) Biotin-labeled cirSLC22A3 probe could pull down miR-19b-3p. (**H**) Ago2 antibody could significantly enrich circSLC22A3 and miR-19b-3p. (**I, J**) MiR-19b-3p mimic inhibited the effects of circSLC22A3 on the invasion and migration ability of TE-1 and KYSE30 cells. Scale bar, 50 μm, 100 μm, 200 μm. *, *P* < 0.05. **, *P* < 0.01. ***, *P* < 0.001. ****, *P* < 0.0001

The current study demonstrated that circSLC22A3 predominantly localized within the cytoplasm (Fig. 1H and I). Previous research has shown that circRNA located in the cytoplasm could play a biological role by absorbing miRNA via sponges to release the control and regulation of downstream target genes [38]. It was speculated that circSLC22A3 may act as a tumor suppressor in ESCC by regulating the expression of downstream target genes via competitive binding of miRNA. First, the miRNAs that circSLC22A3 may interact with were predicted based on the online databases miRanda and circBank and the increased abundance of miRNAs in ESCC from sequencing data. The results revealed that two miRNAs (miR-19b-3p and miR-671-5p) were overlapped (Fig. 4A). Changes in miRNAs were detected by RT-qPCR after overexpression of circSLC22A3 to verify the aforementioned prediction results. The findings showed that only the expression of miR-19b-3p was significantly decreased i after overexpression of circSLC22A3 (Fig. 4B), while that of miR-671-5p did not exhibit a significant change (Fig. 4C). Thus, it was initially speculated that circSLC22A3 may exert its biological function by competitive inhibition of miR-19b-3p. To verify whether circSLC22A3 and miR-19b-3p can bind directly, their binding sites were predicted and mutagenesis was performed (Fig. 4D). The results of dual-luciferase reporter assay demonstrated that miR-19b-3p mimic inhibited the luciferase activity of circSLC22A3-wild-type (WT), but did not impact the luciferase activity of circSLC22A3-mutant (MUT) (Fig. 4E). Next, immunofluorescence probes were designed for circSLC22A3 and miR-19b-3p, labeled as circSLC22A3 and miR-19b-3p respectively. To detect expression localization in both, the images captured by confocal microscopy indicated that circSLC22A3 were co-localized with miR-19b-3p in the cytoplasm (Fig. 4F). RNA pulldown experiments were conducted employing a biotin-labeled circSLC22A3 probe, which demonstrated that the biotin-labeled cirSLC22A3 probe specifically enriched miR-19-3p compared to the NC (Fig. 4G). Subsequently, anti-argonaut 2 (Ago2) antibody or control IgG was used for RIP assays to pull down circSLC22A3 and miR-19b-3p in ESCC cells, followed by RT-qPCR analysis. The results showed that circSLC22A3 and miR-19b-3p were significantly enriched in the Ago2 IP group compared with the IgG control group (Fig. 4H). These results confirmed that circSLC22A3 bound to miR-19b-3p and that circSLC22A3 could inhibit the expression of miR-19b-3p. Combined with TCGA database, miR-19b-3p was found to be highly expressed in ESCC, and patients with high expression of miR-19b-3p had a worse prognosis than those with low expression of miR-19b-3p (Fig. S2A and S2B). After synthesizing miR-19b-3p mimic and inhibitor and verifying their efficiency (Fig. S2C and S2D), it was demonstrated by cell scratch and Transwell assays, that miR-19b-3p promoted the migration and invasion abilities of ESCC cells (Fig. S2E and S2F). The present study investigated whether miR-19b-3p was involved in the inhibitory effect of circSLC22A3 in ESCC. A rescue experiment was conducted by co-transfecting ESCC cells with circSLC22A3 overexpressing vectors and miR-19b-3p mimic. The effect of circSLC22A3 on TE-1 and KYSE30 cell invasion and migration abilities was suppressed by the miR-19b-3p mimic in the scratch and Transwell assay (Fig. 4I and J).

### miR-19b-3p reduces the tumor suppressive capacity of circSLC22A3 via TRAK2

**Fig. 5:**
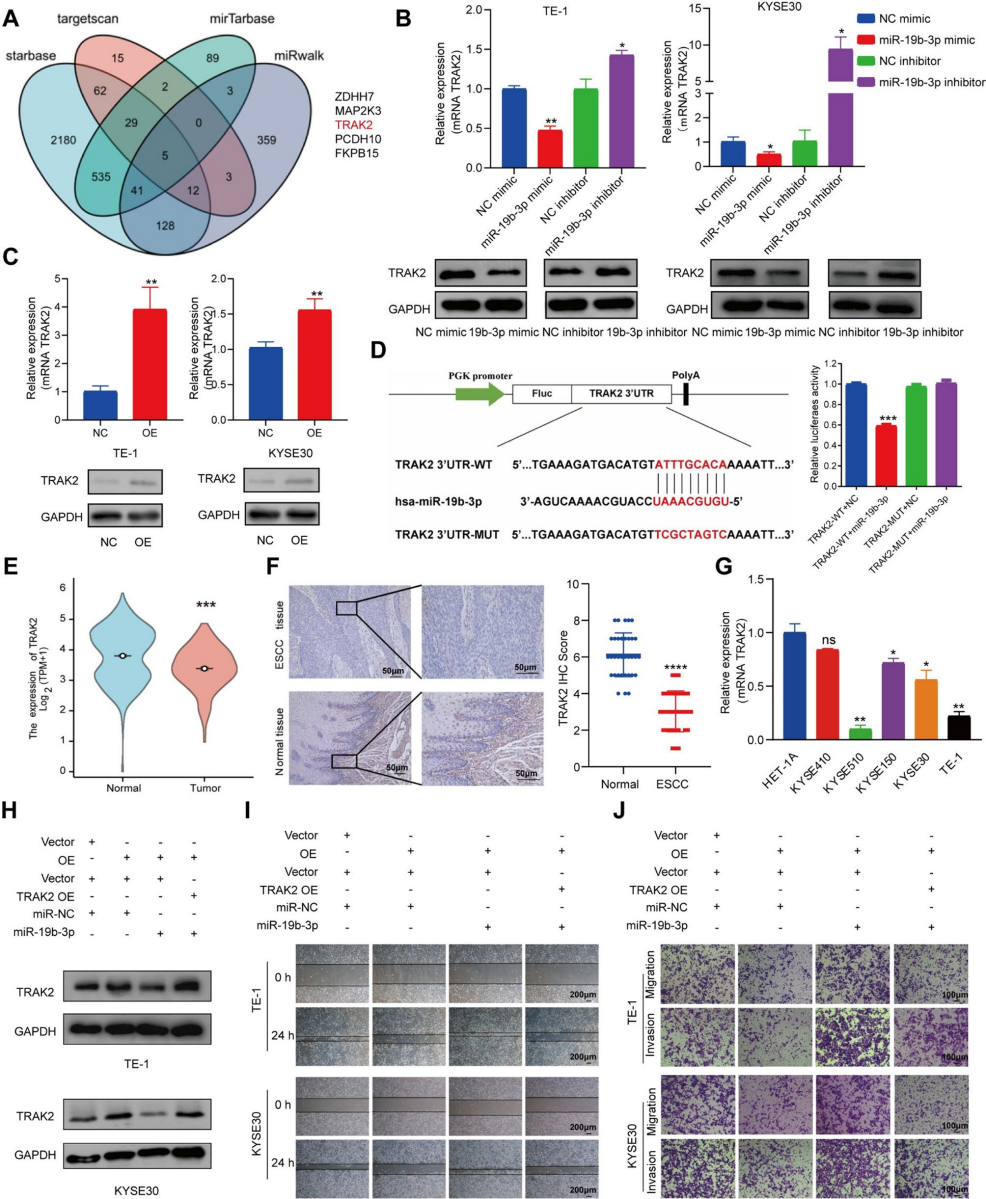
*TRAK2* inhibited tumor development through circSLC22A3/miR-19b-3p axis: (**A**) Venn diagram showed the 5 downstream target genes that miR-19b-3p may bind to. (**B**) MiR-19b-3p inhibited TRAK2 expression as detected by RT-qPCR and Western blot. (**C**) CircSLC22A3 could regulate the expression of TRAK2 in ESCC cells by RT-qPCR and Western blot. (**D**) Dual-luciferase assay was used to verify the binding of miR-19b-3p to *TRAK2*. (**E**) Analysis of the TCGA database revealed low expression of TRAK2 in ESCC tissues. (**F**) ESCC tissues exhibited low expression of TRAK2. (**G**) *TRAK2* expression in ESCC cells lines and the human normal esophageal epithelial cells. (**H**) Western blot assay to detect regulation of TRAK2 protein expression by overexpression of circSLC22A3 or (and) miR-19b-3p mimic. (**I, J**) The overexpression of circSLC22A3 or *TRAK2* effectively suppressed the metastatic promotion induced by miR-19b-3p in cancer cells. Scale bar, 50 μm, 100 μm, 200 μm. *, *P* < 0.05. **, *P* < 0.01. ***, *P* < 0.001

Previous research indicated that circSLC22A3 functioned as a competitive endogenous RNA (ceRNA) by sequestering miR-19b-3p, thereby promoting the migratory and invasive properties of ESCC cells. Therefore, the current study attempted to determine whether circSLC22A3 could affect the progression of ESCC by increasing the expression of the target gene of miR-19b-3p. By employing the TargetScan, miRTarBase, miRWalk, and starBase databases for cross-referencing, five potential downstream target genes of miR-19b-3p (*ZDHHC7*, *MAP2K3*, *TRAK2*, *PCDH10* and *FKBP15*) were successfully identified (Fig. 5A). Considering the negative association between and miR-19b-3p and target genes, the RT-qPCR results showed that only TRAK2 was significantly negatively regulated by miR-19b-3p, which was consistent with the western blot results (Fig. 5B). Meanwhile, overexpression of circSLC22A3 also resulted in up-regulation of TRAK2 expression (Fig. 5C). Similarly, the binding sites of *TRAK2* were predicted and mutated, followed by co-transfection with a miR-19b-3p mimic. Subsequently, a dual luciferase reporter assay was conducted, revealing a significant reduction in luciferase activity in the co-transfected group with *TRAK2*-WT and miR-19b-3p mimic compared with that in the control group. However, no significant alterations were observed in the co-transfected group with *TRAK2*-MUT and miR-19b-3p mimic. The above results, confirmed that circSLC22A3 could regulate *TRAK2* expression via miR-19b-3p (Fig. 5D).

Further investigation on the role of *TRAK2* in ESCC was conducted. By interrogating the data obtained from TCGA, it was demonstrated that TRAK2 exhibited a significant downregulation in ESCC tissues (Fig. 5E). Furthermore, immunohistochemistry analysis of cancer and para-cancer tissue sections from 34 patients with ESCC demonstrated a significant down-regulation of TRAK2 (Fig. 5F), while analysis of the patients’ clinicopathological information revealed that the expression level of TRAK2 was significantly higher in patients with highly differentiated ESCC compared with that in patients with poorly and moderately differentiated ESCC, but there was no significant association between the expression of TRAK2 in the tissues of patients with ESCC and patient’s gender, age, stage, or lymph node metastasis (Fig. S3A). *TRAK2* expression was shown to be downregulated in ESCC cell lines relative to normal esophageal epithelial cells by RT-qPCR assay (Fig. 5G). This observation was in argeement with the expression pattern of circSLC22A3 in ESCC. Cell scratch and Transwell assays showed that the migration and invasion abilities of KYSE30 and TE-1 cells were significantly impaired after overexpression of *TRAK2*(Fig. S3B). Next, we performed a series of rescue experiments to clarify whether circSLC22A3 regulates ESCC progression through the circSLC22A3/miR-19b-3p/TRAK2 axis. Among the results of Western blot experiments, circSLC22A3 was shown to regulate the expression of TRAK2 target proteins through miR-19b-3p (Fig. 5H). Scratch assay and Transwell assay showed that in ESCC cells, the enhancement of migration and invasion induced by miR-19b-3p mimics could be counteracted by overexpression of circSLC22A3 or TRAK2 (Fig. 5I and J and S3C).

### CircSLC22A3 regulates ACSBG1 mRNA stability by binding to IGF2BP1, which is dependent on the recognition of m^6^A modifications

**Fig. 6:**
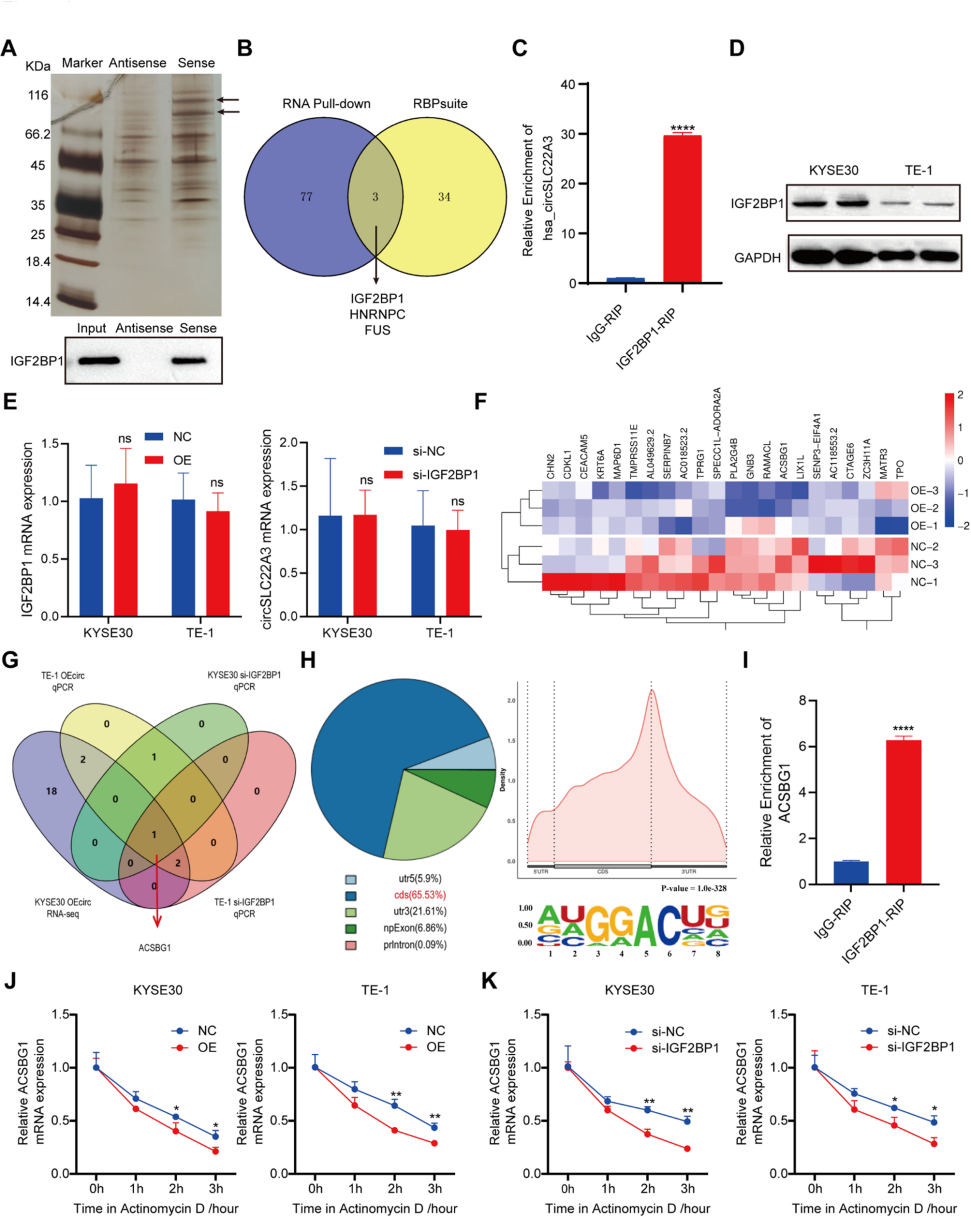
The binding of circSLC22A3 to IGF2BP1 led to a decrease in ACSBG1 mRNA stabilization: (**A**) A schematic of the RNA pulldown experiment for the identification of proteins associated with circSLC22A3. Western blot analysis confirmed that IGF2BP1 was pulled down by the circSLC22A3 probe. (**B**) Venn diagram depicting protein screening results showing the binding of circSLC22A3. (**C**) RIP assay validated the interaction between circSLC22A3 and IGF2BP1. (**D, E**) The expression of circSLC22A3 and *IGF2BP1* in ESCC cells was not influenced by each other. (**F**) Heatmap illustrating alterations in gene enriched by overexpression of circSLC22A3. (**G**) The Venn diagram illustrated the screening of downstream target genes. (**H**) The MeRIP-seq assay revealed that *ACSBG1* was enriched with an m^6^A modification site in the CDS region. (**I**) RIP detection confirmed the interaction between the m^6^A modification site of *ACSBG1* and IGF2BP1. (**J, K**) *ACSBG1* mRNA stability was significantly decreased by circSLC22A3 and *IGF2BP1*. *, *P* < 0.05. **, *P* < 0.01. ****, *P* < 0.0001

Recent studies have shown that circRNAs interact with a variety of proteins and participate in the molecular regulation of various cancer types [23, 24]. In the present study, RNA pull-down and mass spectrometry were used to identify RBPs associated with circSLC22A3 (Fig. 6A). In addition, the intersection with the prediction results of the RBPsuite online platform was employed to screen out three potential binding proteins, namely IGF2BP1, HNRNPC and FUS (Fig. 6B). In the predicted results of RBPsuite, IGF2BP1 and FUS scored higher for binding to circSLC22A3 compared to HNRNPC (Fig. S4A). By further analysis of the human protein map, IGF2BP1 was predominantly localized in the cytoplasm, which was consistent with the cellular localization of circSLC22A3, while FUS was localized in the nucleus (Fig. S4B); thus, subsequent experiments were focused on IGF2BP1. Also, RIP assay confirmed the interaction between IGF2BP1 and circSLC22A3 (Fig. 6C), and RNA pull-down combined with western blot analysis showed the presence of IGF2BP1 in the probe pull-down samples of circSLC22A3 (Fig. 6A). In addition, IGF2BP1 was generally highly expressed in ESCC cells and tumor tissues (Fig. S5A and S5B), while the immunohistochemistry results demonstrated that, in patients with ESCC, the expression level of IGF2BP1 was significantly elevated in patients with poorly and moderately differentiated ESCC compared with that found in patients with highly differentiated ESCC. Moreover, patients with T3 stage had enhanced IGF2BP1 expression compared with that of patients with T2 or T4 stage (Fig. S5C and S5D). Cell functional assays showed that knockdown of IGF2BP1 inhibited the proliferation, migration and invasion of ESCC cells (Fig. S5E-H and S8A-E). Notably, the present study also investigated the association between circSLC22A3 and IGF2BP1, and observed that circSLC22A3 had no obvious effect on either IGF2BP1 protein or mRNA, while knockdown of *IGF2BP1* didn’t alter the expression of circSLC22A3 (Fig. 6D and E). These results suggest that circSLC22A3 may exert its influence on target genes through its interaction with IGF2BP1.

Given the ability of IGF2BP1 to recognize the m^6^A modification on target mRNA and subsequently enhance its stability and translation, the current study aimed to investigate the potential impact of the circSLC22A3/IGF2BP1 complex on target mRNA stability. Through RNA-seq analysis, a significant down-regulation of 22 mRNA transcripts was observed in circSLC22A3 overexpressing KYSE30 cells (Fig. 6F). Subsequently, RT-qPCR validation confirmed a significant decrease in the expression of six genes, including *ACSBG1*, following circSLC22A3 overexpression in TE-1 cells (Fig. S6A). Notably, in both KYSE30 and TE-1 cell lines, only *ACSBG1* mRNA was decreased after knockdown of *IGF2BP1* (Fig. 6G and S6B-C). The selection of *ACSBG1* as a candidate gene for further investigation was based on the following evidence. The result of MeRIP-seq revealed a significant enrichment of *ACSBG1* within the coding sequence region in the m^6^A precipitated fraction, thereby identifying a specific m^6^A modification site sequence for *ACSBG1* (Fig. 6H). RIP assay confirmed the interaction between the m^6^A modification site of *ACSBG1* and IGF2BP1 (Fig. 6I). After overexpressing circSLC22A3 or downregulating the *IGF2BP1* gene, there was a significant decrease in the stability of *ACSBG1* mRNA (Fig. 6J and K).

### *ACSBG1* regulation by circSLC22A3/IGF2BP1 promotes ESCC cell migration and invasion

**Fig. 7:**
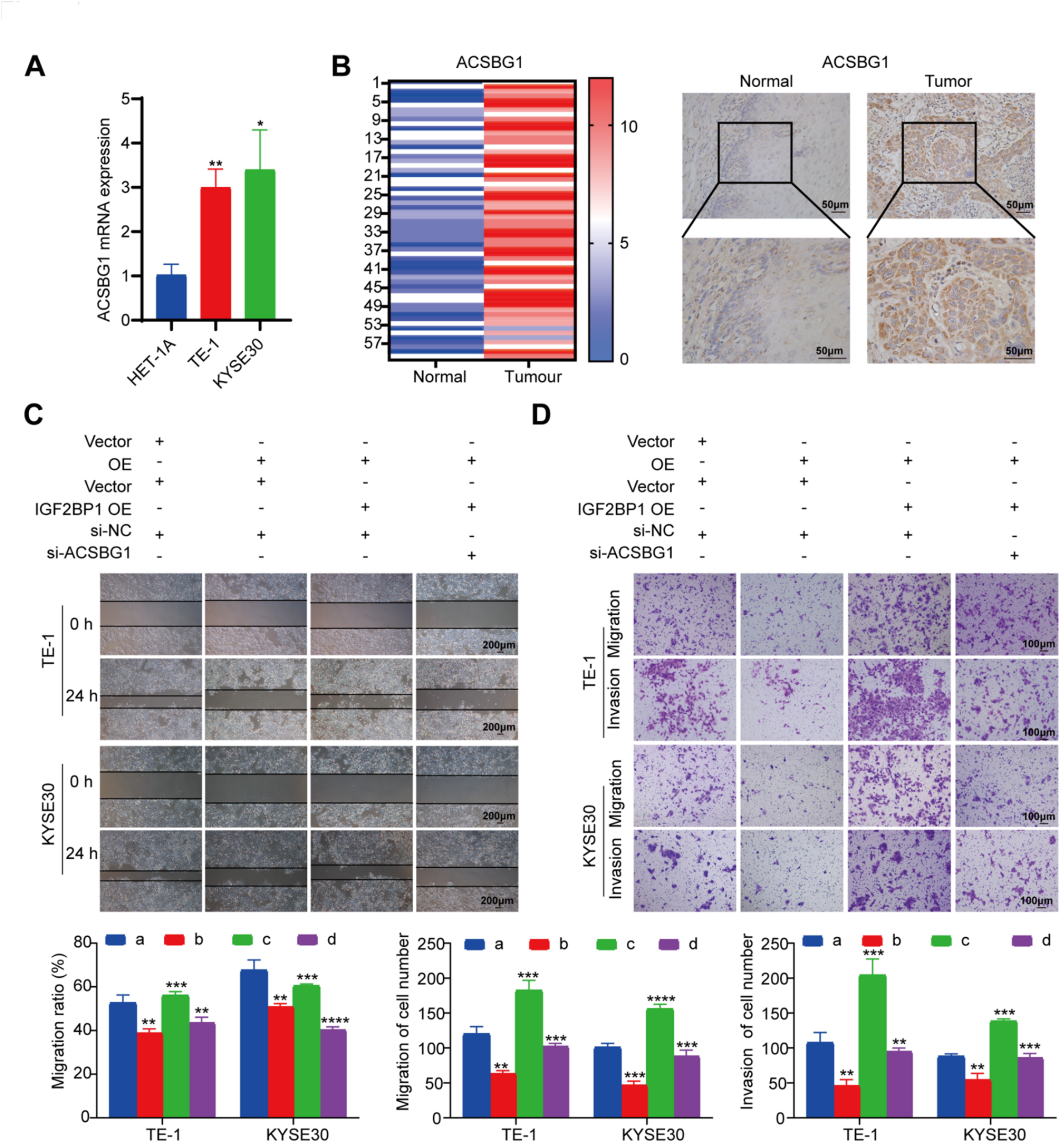
*ACSBG1* regulation by circSLC22A3/IGF2BP1 promotes ESCC cell migration and invasion: (**A, B**) The expression of *ACSBG1* was significantly increased in ESCC tissues and cells. (**C, D**) Overexpression of circSLC22A3 or knockdown of ACSBG1 effectively inhibits the inhibitory effect of overexpression of IGF2BP1 on ESCC cell migration and invasion, group a is NC control, group b is circSLC22A3 OE, group c is circSLC22A3 OE + IGF2BP1 OE, and group d is circSLC22A3 OE + IGF2BP1 OE + si-ACSBG1. *, *P* < 0.05. **, *P* < 0.01. ***, *P* < 0.001. ****, *P* < 0.0001

Considering the reported correlation between *ACSBG1* and breast cancer progression [39], a comprehensive investigation was conducted to determine the potential contribution of *ACSBG1* to ESCC progression. In both ESCC tissues and cells, there was a significantly higher expression of *ACSBG1*, suggesting that it played a role in cancer development (Fig. 7A and B). However, there were no significant differences in ACSBG1 expression in tissues of patients witn ESCC regardless of theirdegree of differentiation, gender, age, stage or lymph node metastasis (Fig. S6D).

After knockdown and overexpression of ACSBG1 in ESCC cells, respectively, the results of Transwell and wound healing assays showed that the migration and invasion abilities of ESCC cells were decreased after knockdown of ACSBG1 and enhanced after overexpression of ACSBG1 (Fig. S7A-D). Notably, ESCC cell proliferation was inhibited after knockdown of ACSBG1, and the opposite was observed after overexpression of ACSBG1 (Fig. S6G), which was further verified by the reduction of tumor size in the nude mouse xenograft model (Fig. S8F-J). These findings underscored the intricate nature of gene regulation. In addition, rescue experiments showed that circSLC22A3 inhibited ESCC cell migration invasion through the IGF2BP1/ACSBG1 axis (Fig. 7C and D).

## Discussion

The incidence of ESCC is relatively high in East Asia, and the majority of patients exhibit local infiltration and distant metastasis at the time of diagnosis, with a poor prognosis and a tendency to relapse after treatments [40]. In recent years, with the advances in medical research, the treatment status of ESCC has been improved to some extent [41–43]; however, the therapeutic effect for patients with advanced ESCC is still not ideal, since ~ 1/3 of patients exhibit tumor recurrence after surgery, and this recurrence is generally difficult to cure. With the advent of the era of precision medicine, molecular targeted therapy has gradually become a research hotspot in the treatment of malignant tumors [44–48].

The prevalence of dysregulated circRNAs in tumors, which have multiple biological functions to regulate tumor progression, has been confirmed by multiple studies [49–51]. In the present study, potential candidate genes were selected through the analysis of our previous sequencing data, and then validated by RT-qPCR. The current study focused on the significantly reduced abundance of circRNA in ESCC, specifically hsa_circ_0078607, which was subsequently designated as circSLC22A3. CircSLC22A3 is the reverse sheared of exon 2, 3, 4 and 5 of the *SLC22A3* gene. Previous research has found that circSLC22A3 inhibits ovarian cancer progression by inducing *Fas* gene expression through sponge adsorption of the oncogenic factor miR-518a-5p, which provides a novel therapeutic approach for ovarian cancer [49]. To date, there is a lack of reports regarding the expression and functional characterization of circSLC22A3 in ESCC. The objective of the present study was to generate a stable cell line overexpressing circSLC22A3 in order to investigate its biological functions in ESCC. In vitro and in vivo experiments demonstrated that circSLC22A3 significantly inhibited the migration and invasion capabilities of ESCC. Previous studies have found that a series of circRNAs have been implicated in the clinicopathology of human cancer [48]. Recent molecular epidemiological studies have found that high circSLC22A3 expression is associated with improved prognosis in ovarian cancer [49]. The current study also confirmed the positive correlation between elevated expression of circSLC22A3 in ESCC and a favorable prognosis, thereby suggesting the potential clinical utility of circSLC22A3 as a diagnostic and prognostic biomarker for ESCC.

CircRNAs are unevenly distributed in cells and possess distinct subcellular localization, which determines its cellular function [51]. Due to the stable localization of circSLC22A3 within the cytoplasm, the present study investigate its potential function as a miRNA sponge, as well as its binding affinity and interaction with proteins. The current results revealed that circSLC22A3 effectively impeded the metastasis of ESCC through its role as a ceRNA for miR-19b-3p. Previous research has consistently demonstrated the involvement of miR-19b-3p in the pathogenesis of diverse diseases and tumorigenesis. In cervical cancer, miR-19b-3p can promote tumor progression by down-regulating *PTEN* [52]. In ESCC, plasma exosome derived miR-19b-3p could inhibit the expression of *MAP2K3* and promote the occurrence and development of ESCC [53]. In the present study, *TRAK2* was identified as the target of circSLC22A3/miR-19b-3p based on bioinformatics analysis. *TRAK2* is a protein coding gene involved in the transport of membrane substances to lysosomes. A previous study found that metastatic and aggressive thyroid cancer was associated with the loss of *TRAK2* expression [54]. *TRAK2* regulates the invasiveness of osteosarcoma by forming a signaling axis with miR-487b, and is a potential therapeutic target and prognostic biomarker [55]. The current data suggested that the abundance of *TRAK2* was significantly reduced in ESCC tissues and cells, indicating a role for *TRAK2* in ESCC. Therefore, the present study has revealed the existence of a ceRNA network involving circSLC22A3/miR-19b-3p/*TRAK2*, providing evidence that circSLC22A3 facilitates the upregulation of *TRAK2* via miR-19b-3p, consequently impeding the advancement of ESCC.

The importance of RBPs in regulating post-transcriptional gene expression is paramount. CircRNAs can act as highly absorptive RBPs, thereby altering the splicing pattern of mRNAs, or having an impact on mRNA stability and translation [56]. Previous studies on the role of circSLC22A3 in regulating PBSs, iobserved that the m^6^A-binding protein IGF2BP1 interacted with circSLC22A3 through RNA pull-down assays. IGF2BP1 was predominantly localized in the cytoplasm, and upregulation of IGF2BP1 expression was associated with tumor progression, including liver cancer [57, 58]. However, overexpression of circSLC22A3 did not exert any discernible impact on the status of IGF2BP1. Moreover, as an m^6^A reader protein, IGF2BP1 exhibited m^6^A-dependent recognition and bound to specific mRNA molecules, thereby functioning as a stabilizer and impeding the degradation process of its target mRNA [59]. In the present study, the common target gene of circSLC22A3 and IGF2BP1, ACSBG1, was screened by RNA-seq combined with RT-qPCR. Unexpectedly, it was found that both IGF2BP1 and ACSBG1 could promote the proliferation of ESCC cells in vitro and in vivo. There is no clear function of circSLC22A3 in ESCC cell proliferation in this study, and there may be other molecules that influence the function of IGF2BP1 and ACSBG1 in cell proliferation. It has been shown that circPTPRA binds to IGF2BP1, inhibits the mRNA stability of Myc and FSCN1, and regulates cell proliferation and migration invasion [60]. Therefore, the mechanism of IGF2BP1 and ACSBG1 in cell proliferation needs further in-depth study and will be our next research direction. Binding of circSLC22A3 to IGF2BP1, which was dependent on its recognition of m^6^A modifications, reduced the mRNA stability of ACSBG1 thereby inhibiting ESCC progression. These results underscore the potential therapeutic value of circRNAs with tumor-suppressing properties, such as circSLC22A3 in the context of future cancer treatments.

The present study was devoted to the dual mechanism of circSLC22A3 in ESCC squamous carcinoma and further examined the expression levels of miR-19b-p, TRAK2 and ACSBG1 in the tissues of the metastatic model mice, and the RT-qPCR results showed that the overexpression of circSLC22A3 resulted in the up-regulation of the expression levels of circSLC22A3 and TRAK2. The expression levels of miR-19b-3p and ACSBG1 decreased, further confirming the findings of this study (Fig. S9).

**Fig. 8:**
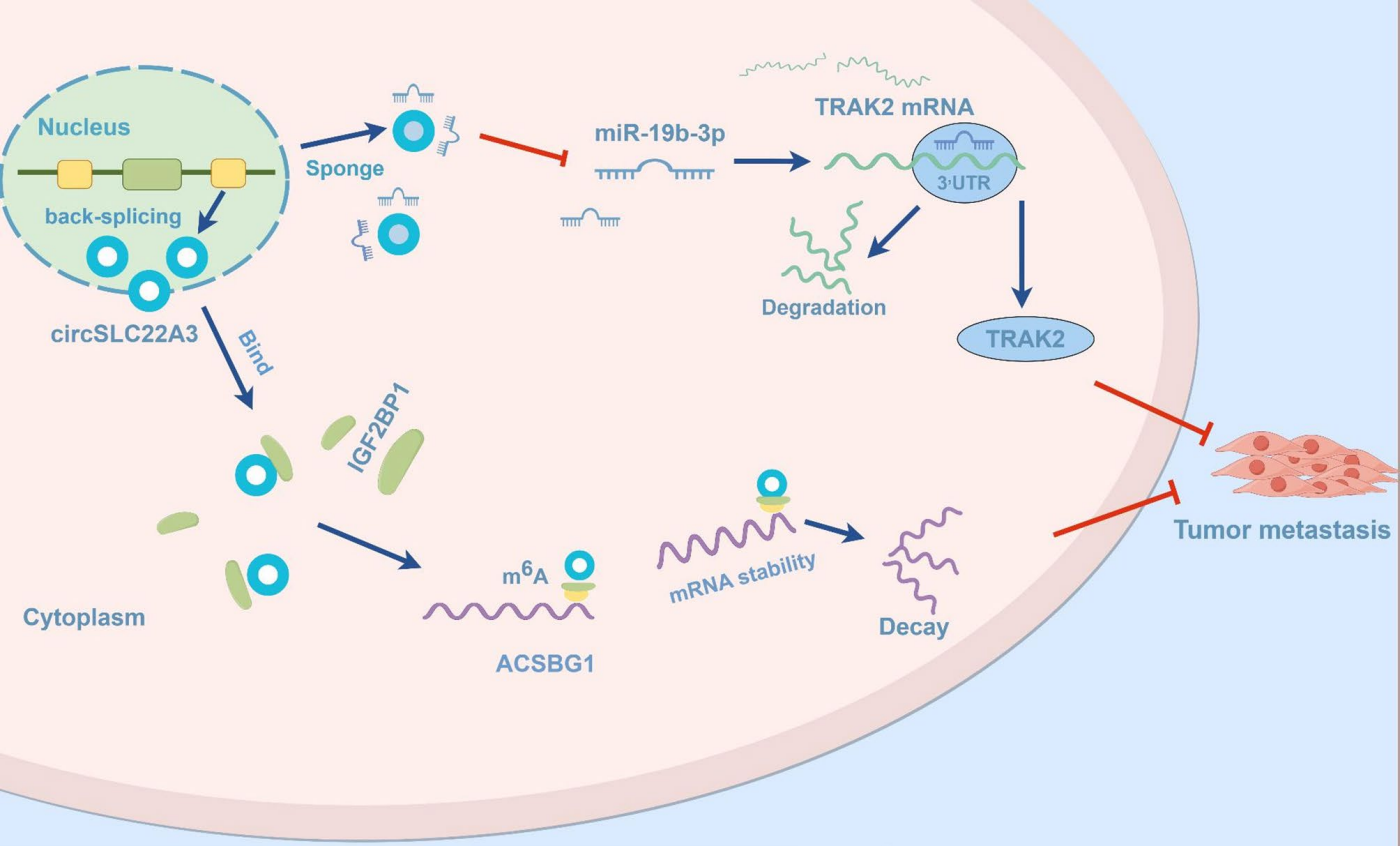
Schematic diagram of the mechanism of double action of CircSLC22A3 in ESCC: CircSLC22A3 suppresses ESCC metastasis through two mechanisms (By Figdraw): firstly, it functions as a miR-19b-3p sponge to upregulate *TRAK2*; secondly, it modulates the stability of *ACSBG1* mRNA in an m^6^A modification-dependent manner by binding with IGF2BP1, leading to downregulation of *ACSBG1* expression

In summary, the present findings revealed that circSLC22A3 was significantly downregulated in ESCC and was predominantly localized in the cytoplasm. It functioned as a RNA by sequestering miRNAs and also interacted with RBPs. Acting as a tumor suppressor, circSLC22A3 exerted a negative regulation on the metastatic potential of ESCC through two mechanisms: Firstly, as a sponge for miR-19b-3p, it enhances the expression of *TRAK2*; secondly, by binding to IGF2BP1, it modulates the stability of *ACSBG1* mRNA in a manner dependent on the recognition of m^6^A modification, thereby downregulating *ACSBG1* expression (Fig. 8). These findings offer novel insights into the metastasis of ESCC and present potential targets for therapeutic intervention.

## Electronic supplementary material

Below is the link to the electronic supplementary material.

Supplementary Material 1

Supplementary Material 2

Supplementary Material 3

## Data Availability

Data are provided in the manuscript or in the Supplementary Information file, where six pairs of transcriptome sequencing data have been uploaded into the GEO database under the dataset code GSE263647.
