# Supplementary material for: CircSLC22A3 inhibits the invasion and metastasis of ESCC via the miR-19b-3p/TRAK2 axis and by reducing the stability of m6A-modified ACSBG1 mRNA
Source: BMC Cancer. 2025 May 30;25:971. doi: 10.1186/s12885-025-14390-8 (PMC12125856; doi:10.1186/s12885-025-14390-8)
Supplement: Supplementary file 1 — Supplementary Material 1 [file 12885_2025_14390_MOESM1_ESM.docx]

Table SⅠ CircSLC22A3 tissue microarray clinical information

| Pathology No. | Clinical stages | Gender | Age | T | N | M |
| --- | --- | --- | --- | --- | --- | --- |
| D08A0181 | 2A | Male | 70 | T3 | N0 | M0 |
| D08A0184 | 3A | Male | 73 | T3 | N1 | M0 |
| D08A0185 | 3 | Female | 74 | T3 | N1 | M0 |
| D08A0187 | 2B | Male | 66 | T3 | N0 | M0 |
| D08A0196 |  | Male | 78 |  | N1 | M0 |
| D08A0201 | 2B | Male | 67 | T2 | N0 | M0 |
| D08A0203 | 3A | Male |  | T3 | N1 | M0 |
| D08A0211 |  | Male | 74 |  | N0 | M0 |
| D08A0214 | 3A | Male | 29 | T3 | N1 | M0 |
| D08A0256 | 3 | Male | 83 | T4 | N0 | M0 |
| D08A0257 | 2 | Male | 65 | T3 | N0 | M0 |
| D08A0260 | 2 | Male | 75 | T3 | N0 | M0 |
| D08A0284 | 2B | Male | 51 | T3 | N0 | M0 |
| D08A0286 |  | Male | 73 |  | N0 | M0 |
| D08A0373 | 3C | Male | 64 | T3 | N3 | M0 |
| D08A0376 | 2B | Male | 64 | T3 | N0 | M0 |
| D08A0380 | 3A | Male | 52 | T3 | N1 | M0 |
| D08A0381 | 3C | Male | 59 | T3 | N3 | M0 |
| D08A0383 | 3A | Male | 70 | T3 | N1 | M0 |
| D08A0385 | 2B | Male | 63 | T2 | N0 | M0 |
| D08A0401 | 3A | Male | 53 | T3 | N1 | M0 |
| D08A0402 | 2A | Female | 71 | T3 | N0 | M0 |
| D08A0403 | 3A | Male | 69 | T3 | N1 | M0 |
| D08A0404 | 3A | Male | 61 | T3 | N1 | M0 |
| D08A0405 | 2 | Male | 77 | T3 | N0 | M0 |
| D08A1772 | 3A | Male | 64 | T3 | N1 | M0 |
| D08A0514 | 3A | Male | 53 | T3 | N1 | M0 |
| D08A0515 | 2B | Male | 66 | T2 | N0 | M0 |
| D08A0571 | 2A | Female | 56 | T3 | N0 | M0 |
| D08A0572 | 2 | Female | 68 | T3 | N0 | M0 |
| D08A1794 |  | Male | 56 |  |  | M0 |
| D08A0577 | 2B | Male | 57 | T2 | N1 | M0 |
| D08A0579 | 2 | Male | 61 | T3 | N0 | M0 |
| D08A0580 | 3C | Male | 68 | T3 | N3 | M0 |
| D08A0581 | 2A | Female | 72 | T3 | N0 | M0 |
| D08A0583 | 2B | Male | 74 | T3 | N0 | M0 |
| D08A0584 | 3A | Male | 72 | T2 | N2 | M0 |
| D08A0588 | 2B | Female | 65 | T3 | N0 | M0 |
| D08A0594 | 2 | Female | 58 | T3 | N0 | M0 |
| D08A0596 | 2B | Female | 63 | T3 | N0 | M0 |
| D08A0649 | 2 | Male | 76 | T3 | N0 | M0 |
| D08A0692 | 3C | Male | 79 | T2 | N3 | M0 |
| D08A0694 | 3A | Male | 72 | T3 | N1 | M0 |
| D08A0696 | 3A | Male | 54 | T3 | N1 | M0 |
| D08A0699 | 3C | Male | 65 | T3 | N3 | M0 |
| D08A1820 | 3A | Male | 62 | T3 | N1 | M0 |
| D08A0718 | 3A | Male | 64 | T3 | N1 | M0 |
| D08A0719 | 1 | Female | 71 | T1 | N0 | M0 |
| D08A0746 | 1 | Female | 74 | T1 | N0 | M0 |
| D08A0748 | 1 | Male | 63 | T1 | N0 | M0 |
| D08A0753 | 3A | Male | 52 | T3 | N1 | M0 |
| D08A1837 | 2B | Male | 73 | T2 | N0 | M0 |
| D08A0205 | 3B | Female | 79 | T3 | N2 | M0 |
| D08A0212 | 2 | Male | 78 | T3 | N0 | M0 |
| D08A0216 | 3B | Male | 84 | T3 | N2 | M0 |
| D08A0218 | 3 | Male | 74 |  | N2 | M0 |
| D08A0334 | 3B | Male | 72 | T3 | N2 | M0 |
| D08A0337 | 3B | Male | 71 | T3 | N2 | M0 |
| D08A0340 | 2B | Male | 51 | T2 | N0 | M0 |
| D08A0593 | 2B | Male | 75 | T3 | N0 | M0 |
| D08A0648 | 3B | Female | 73 | T3 | N2 | M0 |
| D08A0652 | 2B | Female | 79 | T3 | N0 | M0 |
| D08A0702 | 3A | Male | 81 | T3 | N1 | M0 |
| D08A0705 | 2A | Female | 74 | T3 | N0 | M0 |
| D08A0723 | 3A | Female | 68 | T3 | N1 | M0 |
| D08A0188 | 2B | Male | 73 | T3 | N0 | M0 |
| D08A0209 | 3A | Male | 72 | T3 | N1 | M0 |
| D08A0219 | 3A | Male | 66 | T3 | N1 | M0 |
| D08A0290 | 3A | Male | 63 | T3 | N1 | M0 |
| D08A0651 | 3A | Female | 57 | T3 | N1 | M0 |
| D08A0650 | 3A | Male | 61 | T3 | N1 | M0 |
| D08A1844 | 3A | Female | 51 | T3 | N1 | M0 |
| D08A0221 |  | Male | 50 |  | N0 | M0 |
| D08A0285 | 3B | Male | 51 | T3 | N2 | M0 |
| D08A0294 | 3B | Male | 60 | T3 | N2 | M0 |
| D08A0336 | 2B | Female | 62 | T2 | N0 | M0 |
| D08A0391 | 2B | Male | 73 | T3 | N0 | M0 |
| D08A0394 | 2B | Male | 51 | T3 | N0 | M0 |
| D08A0395 | 3B | Male | 49 | T3 | N2 | M0 |
| D08A0400 | 3A | Male | 48 | T3 | N1 | M0 |
| D08A0477 | 3A | Male | 61 | T3 | N1 | M0 |
| D08A0510 | 2 | Male | 65 | T3 | N0 | M0 |
| D08A0548 | 1 | Female | 63 | T1 | N0 | M0 |
| D08A0582 | 2A | Male | 57 | T3 | N0 | M0 |
| D08A0589 | 3A | Male | 65 | T3 | N1 | M0 |
| D08A0595 | 3A | Male | 80 | T3 | N1 | M0 |
| D08A0655 | 1B-2 | Female | 71 | T3 | N0 | M0 |
| D08A0659 | 3B | Male | 50 | T3 | N2 | M0 |
| D08A0660 | 3A | Male | 54 | T2 | N2 | M0 |
| D08A1822 | 3B | Male | 59 | T3 | N2 | M0 |
| D08A0803 | 2B | Male | 52 | T2 | N1 | M0 |
| D08A0804 | 2 | Female | 74 | T3 | N0 | M0 |
| D08A0724 | 3B | Male | 74 | T3 | N2 | M0 |
| D08A0745 | 3A | Male | 79 | T3 | N1 | M0 |
| D08A0752 | 3A | Male | 65 | T3 | N1 | M0 |
| D08A0807 | 2B | Female | 69 | T2 | N0 | M0 |
| D08A0834 | 2B | Male | 77 | T2 | N0 | M0 |

Table SⅡ Correlation between circSLC22A3 expression and clinicopathological characteristics

|  | variables | CircSLC22A3 expression | | p value |
| --- | --- | --- | --- | --- |
|  |  | low | high |  |
| Age (year) |  | 65 | 65.5 | 0.937 |
| Sex |  |  |  | 0.517 |
|  | Male | 26 | 49 |  |
|  | Female | 6 | 16 |  |
| Size (cm) |  | 5 | 5 | 0.436 |
| Grade |  |  |  | 0.490 |
|  | I/II | 27 | 51 |  |
|  | III | 5 | 14 |  |
| T stage |  |  |  | 0.136 |
|  | T1/T2 | 3 | 14 |  |
|  | T3/T4 | 27 | 47 |  |
| N stage |  |  |  | 0.885 |
|  | N0 | 15 | 29 |  |
|  | N1 | 17 | 35 |  |
| TNM stage |  |  |  | 0.892 |
|  | Ι/II | 14 | 28 |  |
|  | III/IV | 16 | 34 |  |

Table SⅢ TRAK2 clinical information

| Pathology No. | Tumor differentiation | Gender | Age | T | N | M |
| --- | --- | --- | --- | --- | --- | --- |
| 2020-011421 | low | Male | 47 | T4 | N3 | M1 |
| 2020-015115 | high | Male | 68 | T3 | N0 | M0 |
| 2020-014695 | high | Male | 67 | T3 | N0 | M0 |
| 2020-014296 | high | Male | 66 | T1 | N0 | M0 |
| 2020-013935 | high | Male | 79 | T3 | N1 | M1 |
| 2020-011898 | high | Male | 68 | T2 | N0 | M0 |
| 2019-008629 | high | Female | 67 | T2 | N0 | M0 |
| 2021-003424 | high | Male | 72 | T2 | N0 | M0 |
| 2020-027153 | moderate | Male | 65 | T3 | N2 | M1 |
| 2020-023978 | high | Male | 70 | T2 | N0 | M0 |
| 2020-015310 | low | Male | 69 | T1 | N1 | M0 |
| 2020-018674 | moderate | Male | 72 | T2 | N1 | M0 |
| 2020-013232 | moderate | Male | 67 | T2 | N0 | M0 |
| 2020-021451 | moderate | Male | 66 | T2 | N2 | M0 |
| 2020-020130 | moderate | Male | 67 | T1 | N0 | M0 |
| 2020-018529 | moderate | Female | 76 | T1 | N0 | M0 |
| 2020-018362 | moderate | Male | 51 | T3 | N3 | M1 |
| 2020-009676 | moderate | Female | 75 | T3 | N1 | M1 |
| 2021-004574 | moderate | Male | 66 | T1 | N0 | M0 |
| 2020-029490 | moderate | Female | 69 | T1 | N0 | M0 |
| 2020-027975 | moderate | Female | 69 | T2 | N0 | M0 |
| 2020-027607 | moderate | Female | 75 | T3 | N1 | M0 |
| 2020-027484 | moderate | Female | 69 | T1 | N0 | M0 |
| 2020-026080 | moderate | Female | 71 | T2 | N0 | M0 |
| 2020-024696 | moderate | Male | 71 | T2 | N2 | M1 |
| 2020-020279 | moderate | Male | 70 | T1 | N1 | M0 |
| 2020-017653 | moderate | Female | 74 | T1 | N1 | M1 |
| 2020-011658 | high | Male | 68 | T3 | N2 | M1 |
| 2020-025026 | high | Male | 73 | T3 | N2 | M1 |
| 2020-016008 | high | Male | 68 | T3 | N1 | M1 |
| 2020-026864 | moderate | Female | 78 | T2 | N0 | M0 |
| 2020-025287 | moderate | Male | 78 | T3 | N21529 | M1 |
| 2020-024566 | moderate | Male | 70 | T3 | N0 | M0 |
| 2022-011529 | high | male | 62 | T3 | N1 | M0 |

Table SⅣ IGF2BP1 clinical information

| Pathology No. | Tumor differentiation | Gender | Age | T | N | M |
| --- | --- | --- | --- | --- | --- | --- |
| 2022-002917 | low | Male | 75 | T4 | N0 | M0 |
| 2021-003320 | low | Male | 76 | T2 | N1 | M0 |
| 2020-011421 | low | Male | 47 | T2 | N2 | M0 |
| 2022-012923 | low | Male | 76 | T3 | N2 | M0 |
| 2020-013537 | low | Female | 62 | T4 | N2 | M0 |
| 2020-020686 | low | Male | 71 | T3 | N0 | M0 |
| 2020-021503 | low | Male | 71 | T3 | N2 | M0 |
| 2020-025486 | low | Male | 74 | T3 | N0 | M0 |
| 2020-026414 | low | Male | 81 | T3 | N1 | M0 |
| 2020-018674 | low | Male | 72 | T1 | N1 | M0 |
| 2021-005693 | low | Female | 80 | T1 | N0 | M0 |
| 2020-018529 | moderate | Female | 76 | T1 | N0 | M0 |
| 2020-020056 | moderate | Female | 71 | T2 | N0 | M0 |
| 2020-013232 | moderate | Male | 67 | T1 | N0 | M0 |
| 2020-016101 | moderate | Female | 70 | T3 | N1 | M0 |
| 2020-027975 | moderate | Male | 69 | T2 | N0 | M0 |
| 2020-012671 | moderate | Male | 59 | T4 | N1 | M0 |
| 2020-026080 | moderate | Female | 71 | T2 | N0 | M0 |
| 2020-013443 | moderate | Female | 76 | T3 | N1 | M0 |
| 2020-018362 | moderate | Male | 51 | T4 | N3 | M0 |
| 2021-004574 | moderate | Male | 66 | T1 | N0 | M0 |
| 2020-025026 | high | Male | 73 | T3 | N2 | M0 |
| 2020-014695 | high | Male | 67 | T4 | N0 | M0 |
| 2020-020279 | Moderate-high | Male | 70 | T1 | N1 | M0 |
| 2020-027153 | High-moderate | Male | 65 | T3 | N2 | M0 |
| 2020-011529 | high | Male | 62 | T4 | N1 | M0 |
| 2019-008629 | high | Female | 67 | T3 | N0 | M0 |
| 2020-011898 | high | Male | 68 | T4 | N0 | M0 |
| 2020-013122 | high | Male | 68 | T3 | N0 | M0 |
| 2020-015639 | high | Male | 71 | T1 | N0 | M0 |
| 2020-023978 | high-moderate | Male | 70 | T2 | N0 | M0 |

Table SⅤ ACSBG1 clinical information

| Pathology No. | Tumor differentiation | Gender | Age | T | N | M |
| --- | --- | --- | --- | --- | --- | --- |
| 2022-002658 | low | Female | 72 | T4 | N1 | M1 |
| 2022-002917 | low | Male | 75 | T4 | N0 | M0 |
| 2022-012923 | low | Male | 76 | T3 | N2 | M0 |
| 2021-003320 | low | Male | 76 | T2 | N1 | M0 |
| 2022-015010 | low | Male | 65 | T4 | N3 | M1 |
| 2020-011421 | low | Male | 47 | T2 | N2 | M0 |
| 2020-013537 | low | Female | 62 | T4 | N2 | M0 |
| 2020-018674 | low | Male | 72 | T1 | N1 | M0 |
| 2020-020058 | low | Female | 55 | T3 | N2 | M0 |
| 2020-021503 | low | Male | 71 | T3 | N2 | M0 |
| 2020-025486 | low | Male | 74 | T3 | N0 | M0 |
| 2020-026414 | low | Male | 81 | T3 | N1 | M0 |
| 2020-015310 | low | Male | 69 | T1 | N1 | M0 |
| 2021-001233 | low | Male | 74 | T4 | N3 | M1 |
| 2021-002519 | low | Female | 80 | T2 | N2 | M0 |
| 2021-004741 | low | Female | 53 | T1 | N1 | M0 |
| 2022-003789 | low | Male | 71 | T3 | N1 | M0 |
| 2022-004936 | low | Male | 74 | T3 | N1 | M0 |
| 2020-014555 | moderate | Male | 70 | T4 | N3 | M1 |
| 2020-018054 | moderate | Male | 65 | T4 | N2 | M0 |
| 2020-013232 | moderate | Male | 67 | T1 | N0 | M0 |
| 2020-009676 | moderate | Female | 75 | T3 | N1 | M0 |
| 2021-004574 | moderate | Male | 66 | T1 | N0 | M0 |
| 2019-001860 | moderate | Male | 66 | T4 | N1 | M0 |
| 2020-020686 | moderate | Male | 71 | T3 | N0 | M0 |
| 2020-026080 | moderate | Female | 71 | T2 | N0 | M0 |
| 2020-012671 | moderate | Male | 59 | T4 | N1 | M0 |
| 2021-000302 | moderate | Male | 66 | T3 | N2 | M1 |
| 2020-024062 | moderate | Female | 70 | T1 | N0 | M0 |
| 2019-021106 | moderate | Male | 65 | T2 | N0 | M0 |
| 2021-004262 | moderate | Male | 73 | T4 | N1 | M1 |
| 2019-004753 | moderate | Female | 63 | T3 | N0 | M0 |
| 2019-004945 | moderate | Male | 66 | T3 | N2 | M0 |
| 2019-007451 | moderate | Female | 69 | T2 | N0 | M0 |
| 2020-016101 | moderate | Female | 70 | T3 | N1 | M0 |
| 2020-018362 | moderate | Male | 51 | T4 | N3 | M0 |
| 2020-018529 | moderate | Female | 76 | T1 | N0 | M0 |
| 2020-013935 | high | Male | 79 | T4 | N1 | M1 |
| 2020-014695 | high | Male | 67 | T4 | N0 | M0 |
| 2020-015252 | high | Male | 63 | T3 | N1 | M0 |
| 2020-015639 | high | Male | 71 | T1 | N0 | M0 |
| 2020-016008 | high | Male | 68 | T3 | N1 | M0 |
| 2019-008629 | high | Female | 67 | T3 | N0 | M0 |
| 2020-018890 | high | Female | 76 | T2 | N0 | M0 |
| 2020-020279 | high | Male | 70 | T1 | N1 | M0 |
| 2020-023978 | high | Male | 70 | T2 | N0 | M0 |
| 2020-011529 | high | Male | 62 | T4 | N1 | M0 |
| 2020-027153 | high | Male | 65 | T3 | N2 | M0 |
| 2019-002912 | high | Male | 73 | T3 | N2 | M1 |
| 2019-004718 | high | Female | 67 | T4 | N0 | M0 |
| 2019-006311 | high | Female | 55 | T4 | N2 | M1 |
| 2019-007738 | high | Female | 66 | T4 | N0 | M0 |
| 2020-015818 | high | Male | 74 | T2 | N1 | M0 |
| 2020-018550 | high | Male | 68 | T3 | N1 | M0 |
| 2019-020749 | high | Male | 61 | T2 | N0 | M0 |
| 2020-023666 | high | Male | 62 | T4 | N1 | M0 |
| 2019-000611 | high | Male | 69 | T2 | N0 | M0 |
| 2020-011898 | high | Male | 68 | T4 | N0 | M0 |
| 2020-013122 | high | Male | 68 | T3 | N0 | M0 |
| 2020-015251 | high-moderate | Male | 62 | T3 | N0 | M0 |

Table SⅥ Mimic, inhibitor, siRNAs and shRNAs sequences

| Name | Sequence（5'-3'） |
| --- | --- |
| hsa-miR-19b-3p inhibitor | UCAGUUUUGCAUGGAUUUGCACA |
| hsa-miR-19b-3p mimic | UGUGCAAAUCCAUGCAAAACUGAAGUUUUGCAUGGAUUUGCACAUU |
| shIGF2BP1 and si-IGF2BP1#1 | sense: CGGGAAAGUAGAAUUACAAGG |
|  | antisense: UUGUAAUUCUACUUUCCCGGA |
| si-IGF2BP1#2 | sense: CCUGAAGAAGGUAGAGCAAGA |
|  | antisense: UUGCUCUACCUUCUUCAGGUU |
| shACSBG1 and si-ACSBG1#1 | sense: GGCUGUACAGCUCAGGCAAGU |
|  | antisense: UUGCCUGAGCUGUACAGCCGG |
| si-ACSBG1#2 | sense: AGUGCUGUGUGCUAGUCUACA |
|  | antisense: UAGACUAGCACACAGCACUGG |
| si-NC and shNC | sense: UUCUCCGAACGUGUCACGUTT  antisense: ACGUGACACGUUCGGAGAATT |

Table SⅦ Probe sequences

| Gene name | Probe sequences | Label |
| --- | --- | --- |
| circSLC22A3 probe1 | GTCAAAC+TCTGAG+TAAT+TTGATGAG | 5'Biotin |
| circSLC22A3 probe2 | CAAGG+TCAAACTC+TGAGTAAT+TTGA | 5'Biotin |
| circSLC22A3 probe3 | CACAGACAAGG+TCAAAC+TCTGAG+TA | 5'Biotin |
| miR-19b-3p | TCAGT+TTTGCATGGAT+TTGCACA | 5'CY3 |

Table SⅧ The primer sequence of RT-qPCR and RIP

| Gene | Primer sequence (5'‑3') |
| --- | --- |
| GAPDH | F: GGAGTCCACTGGCGTCTTCA |
|  | R: GTCATGAGTCCTTCCACGATACC |
| CircSLC22A3 | F: CATCAAATTACTCAGAGTTTGAC |
|  | R: AGGATAGCAAGTAAATGACGAT |
| SLC22A3 | F: ACTTGCTATCCTGCCTTGGT |
|  | R: CACGTAGCAAGTCATCCACG |
| miR-19b-3p | F: CGTGTGCAAATCCATGCAA |
|  | R: AGTGCAGGGTCCGAGGTATT |
| TRAK2 | F: GCCGAACCACTCCAGTCAT |
|  | R: GCTTTGGTTGAGAAGGACAGC |
| miR-671-5p | F: AGGAAGCCCTGGAGGGG |
|  | R: AGTGCAGGGTCCGAGGTATT |
| PCDH10 | F: AGCGGTCCTTTTCCACCTTT |
|  | R: TGCTGCTTCAGGTAAGTCCT |
| FKBP15 | F: TTGGTTCCAAGGGCTGTCTG |
|  | R: ACAGAACCATCATGCCCCAG |
| ZDHHC7 | F: TGTTTTCAGGGGGTGCCTTT |
|  | R: AGCAACAGTACTCACTGGGC |
| MAP2K3 | F: CCCCAGTCCAAAGGAAAATCC |
|  | R: TCTACCACCCCATAGGCTCC |
| IGF2BP1 | F: GCGGCCAGTTCTTGGTCAA |
|  | R: TTGGGCACCGAATGTTCAATC |
| ACSBG1 | F: ACACTGTGCATCGGATGTTCT |
|  | R: AGGAGATGTGTTCCCACTTGT |
| CTAGE6 | F: GGTGACCGCCAGAGCAG |
|  | R: CTCAGGTAGTGCTGCCACAA |
| AC118553.2 | F: TTTCACAAAGACAGACCGCAT |
|  | R: TGCCATACACACTTCCTGGT |
| SENP3-E2F4A1 | F: CCGACCCTCTTTTGATGCCT |
|  | R: AGTGGAATGTCAGCGAGGTG |
| SPECC1L-ADORA2 | F: TCCCCTTTGCCATCACCATC |
|  | R: CAATGTAGCGGTCAATGGCG |
| CEACAM5 | F: CTGTCCAATGACAACAGGACC |
|  | R: ACGGTAATAGGTGTATGAGGGG |
| TMPRSS11E | F: CCTGGCAGTGTGCATTGGA |
|  | R: CAAGTCTCTGGCTCATTTCTGT |
| AL049629.2 | F: GTGACTATGTGGGAAGCCCC |
|  | R: TCCCAAGGCTAATGGCAAGG |
| AC018523.2 | F: TAGTGCGACTCTAAAGCACCC |
|  | R: AAAACCAGGAGCGTGATGTCT |
| ZC3H11A | F: GATGGCCTTTTCCTACCTCCG |
|  | R: GGACTGGACAGACAATTTGTTCT |
| MATR3 | F: ATCAATGGAGCAAGTCACAGTC |
|  | R: TGCAACATGAATGGATCACCC |
| TPO | F: CTGTCACGCTGGTTATGGC |
|  | R: GCTAGAGACACGAGACTCCTCA |
| CHN2 | F: TTCCAAGCACGTTCCCAATGA |
|  | R: CATGGGTCTCTGAGTGTTGTG |
| MAP6D1 | F: GGCTACTCGGACCTCGACA |
|  | R: TCCCGCTGGTACTGAGTGAG |
| KRT6A | F: AGCACTAAAGTGCGTCTGCT |
|  | R: GTGAGCAATGGGTGCTCAGA |
| CDKL1 | F: GAGCATAACTTGGCAGACACT |
|  | R: CGGAATGTTTCGTGATGAGGAT |
| RAMACL | F: TGAGTCAGCTCCACACTTGAG |
|  | R: TTGTGTGCAATTGTCCAGGC |
| TPRG1 | F: TCAGCCTTATATCTCACGGAAGT |
|  | R: TTGAATGGTCTCTCCAGAAGTCT |
| LIX1L | F: GCCCTCCTTATGTCTGCTATGT |
|  | R: ACTCATCAGTGATTCTTCGGGA |
| GNB3 | F: CGGACGTTAAGGGGACACC |
|  | R: CGAGGCACTTACCAGCAGC |
| SERPINB7 | F: AAATGCAGAGTTTTGCTTCAACC |
|  | R: GAAGAGTTTCCATATCCTGAGGC |
| PLA2G4B | F: GACTCAGTCTCATGGCTGTGG |
|  | R: GTTCCAGACAGGGCTACTGC |
| hsa_circ_SLC22A3(RIP) | F: TGATTACTCGGAAGAAAGG |
|  | R: AGACAAGGTCAAACTCTG |
| ACSBG1(RIP) | F: TCTTTTTGATGTGAACTTTCCCAGG |
|  | R: GCACAAAATGACACTCTTCTAAAAC |
