# Supplementary figures and images for: CircSLC22A3 inhibits the invasion and metastasis of ESCC via the miR-19b-3p/TRAK2 axis and by reducing the stability of m6A-modified ACSBG1 mRNA
Source: BMC Cancer. 2025 May 30;25:971. doi: 10.1186/s12885-025-14390-8 (PMC12125856; doi:10.1186/s12885-025-14390-8)

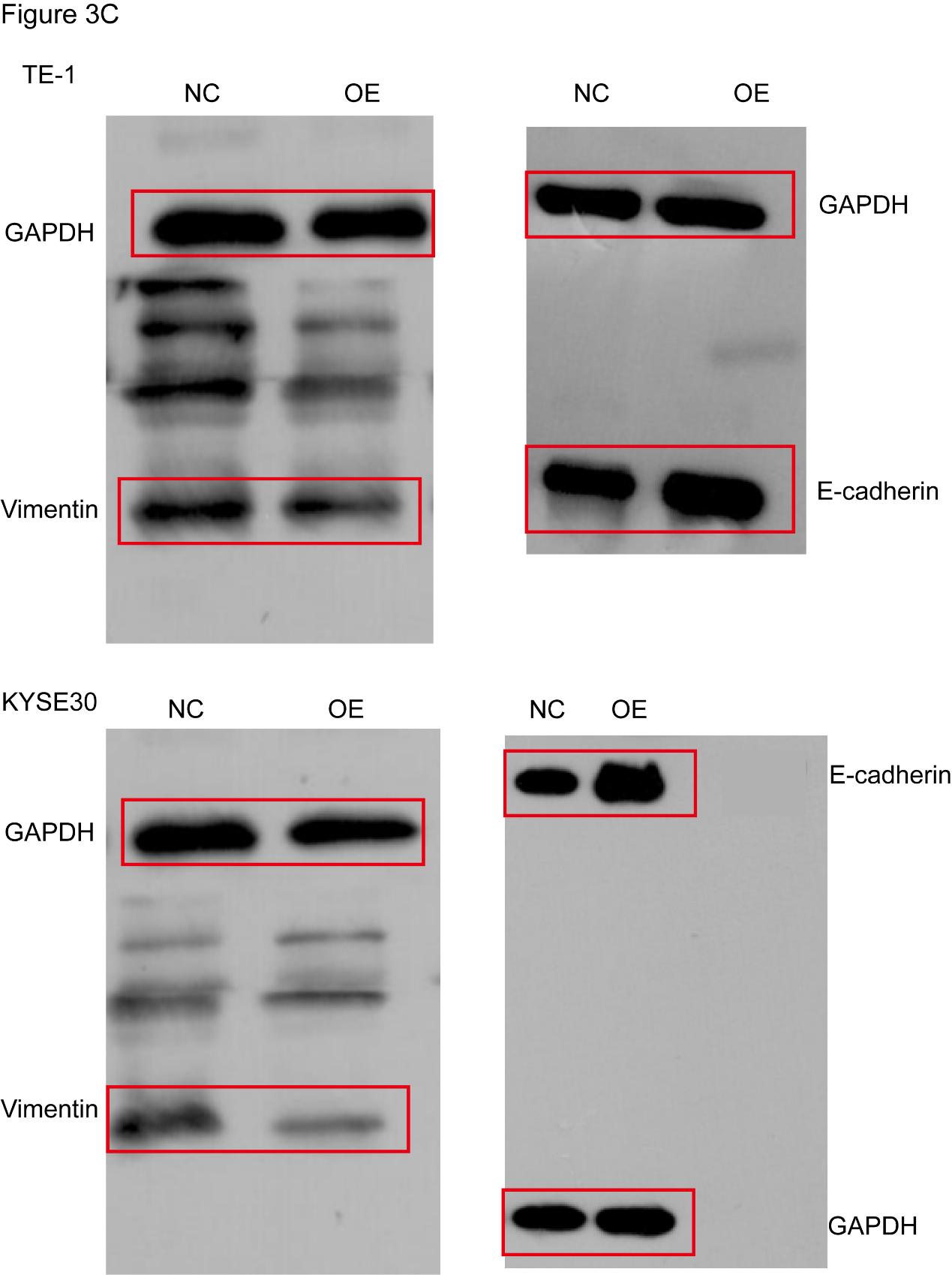

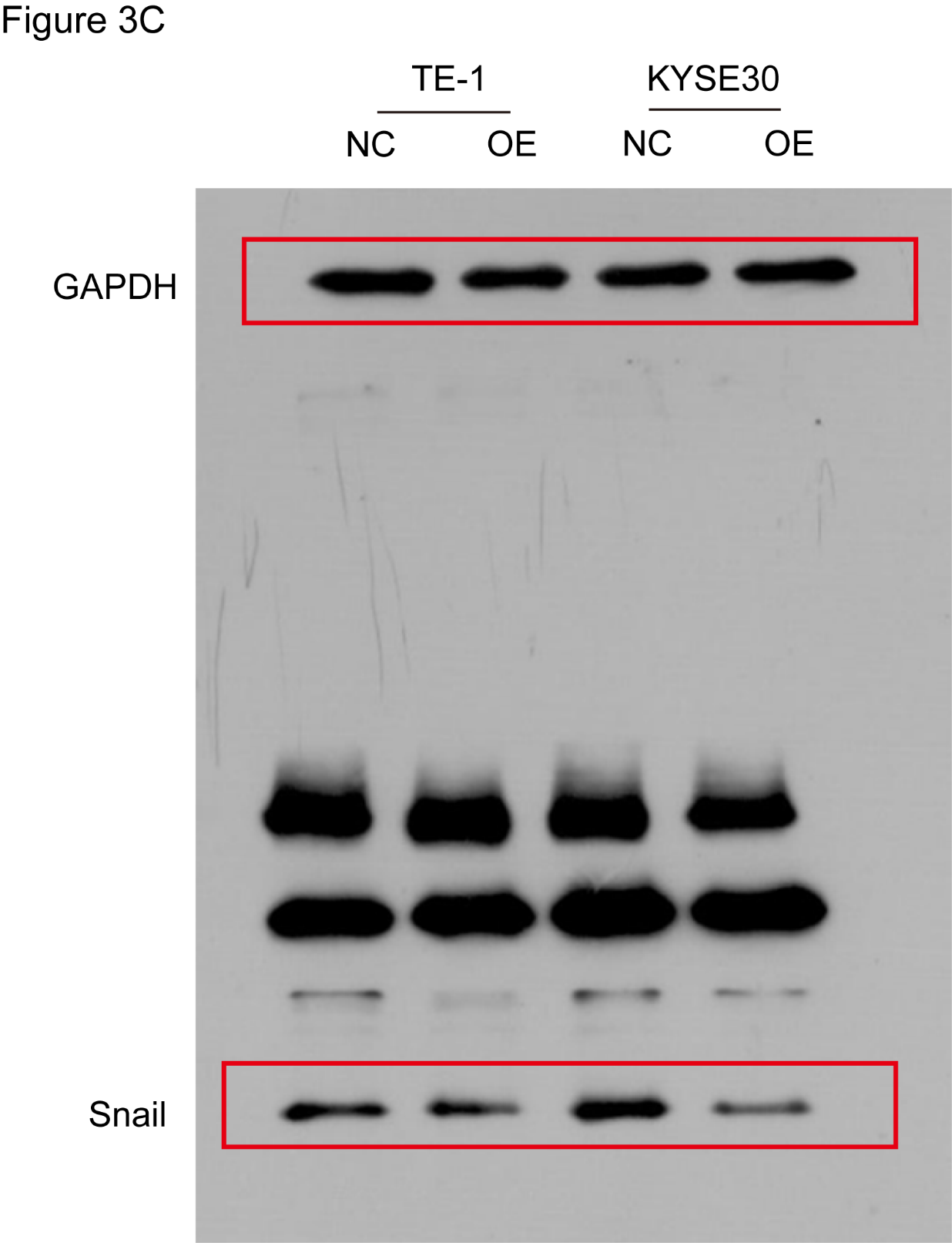


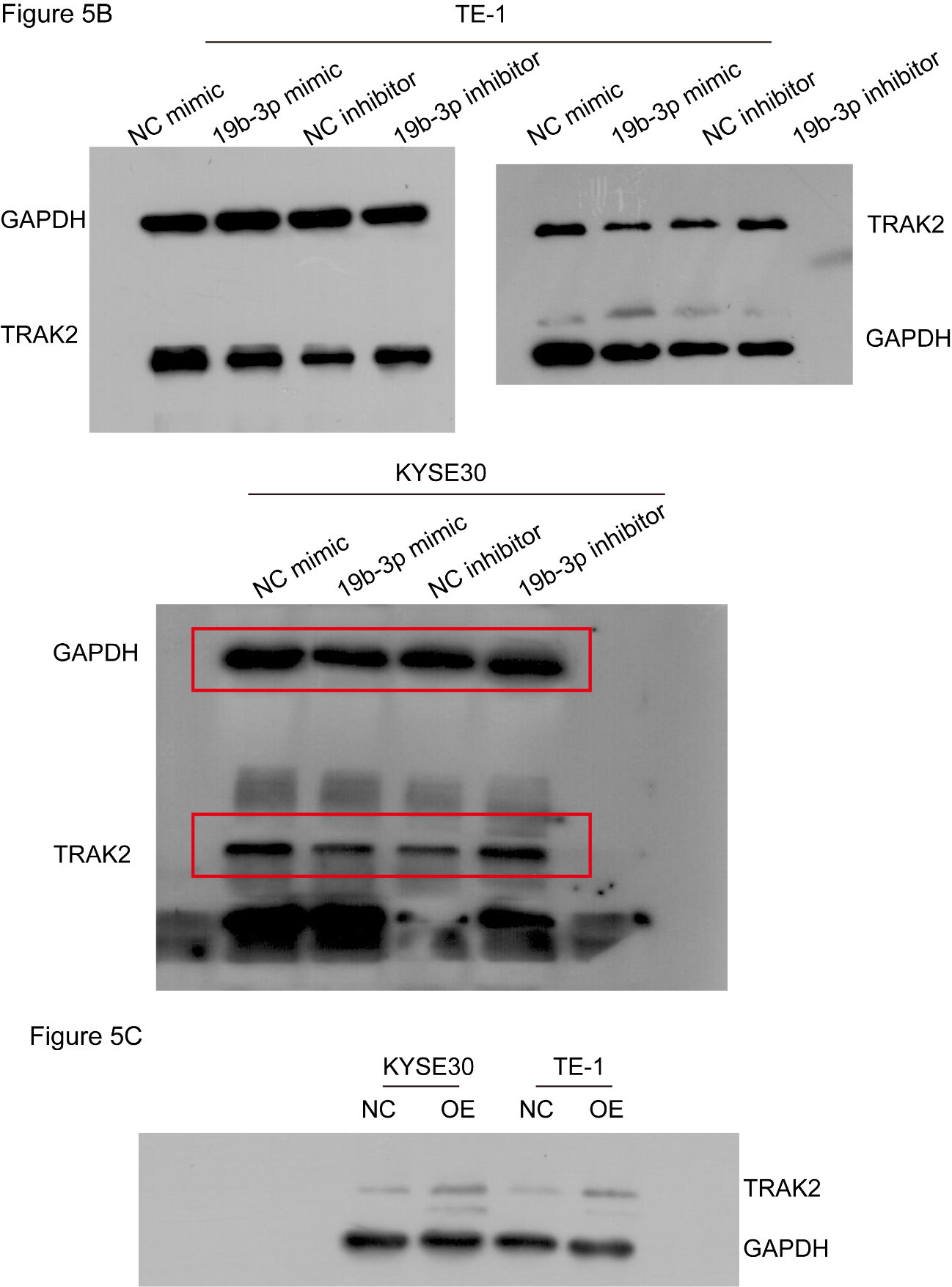


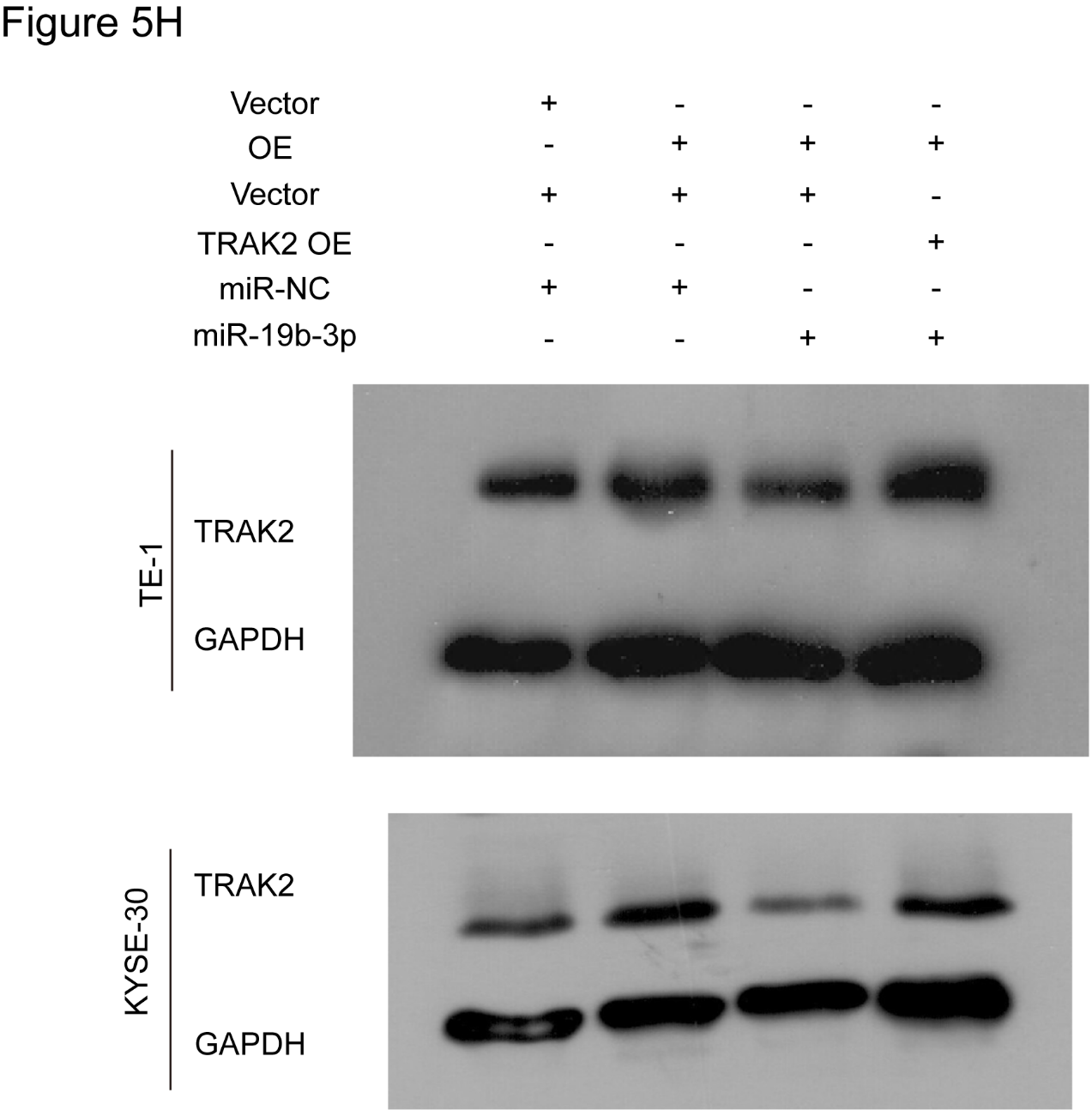


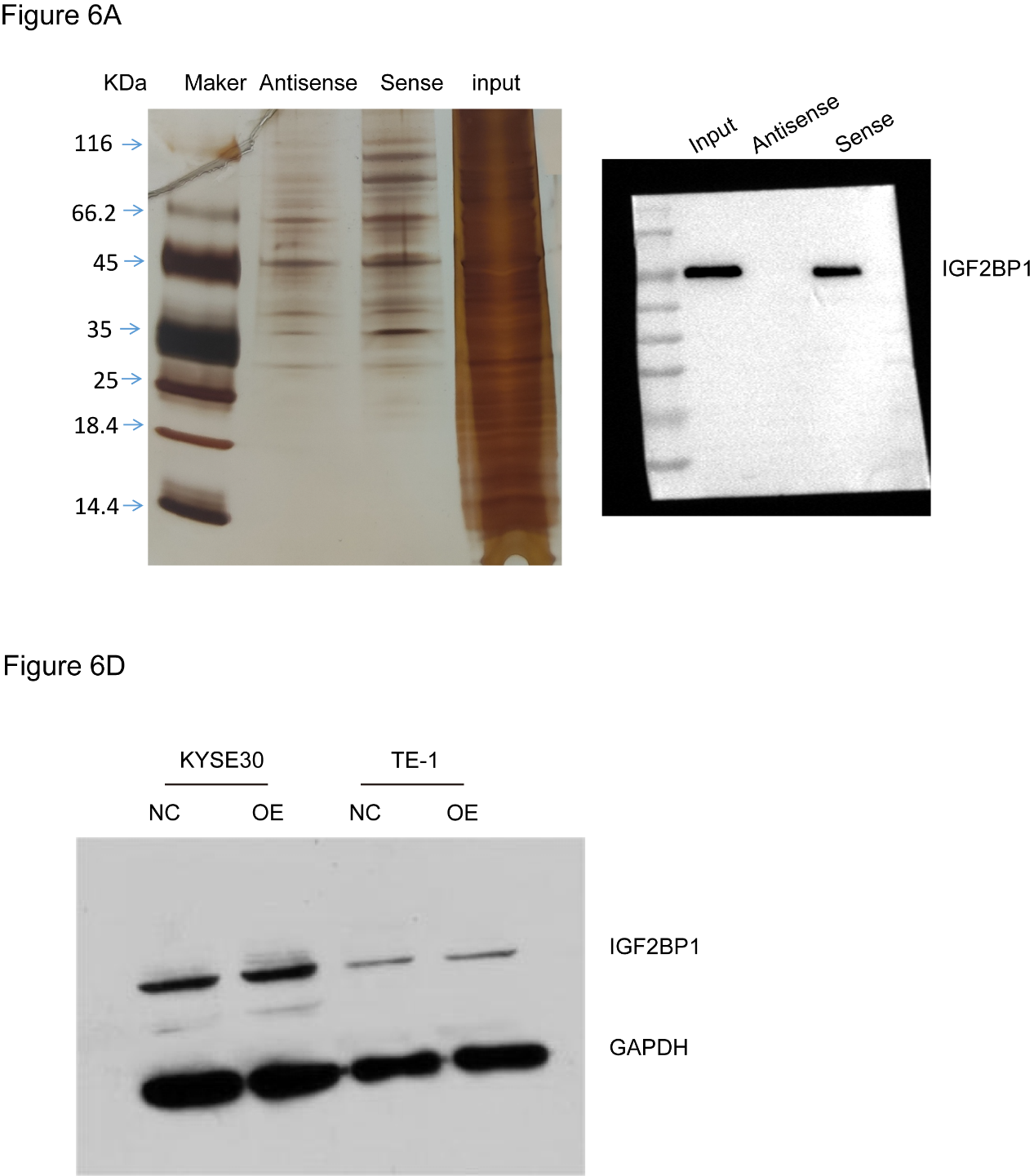

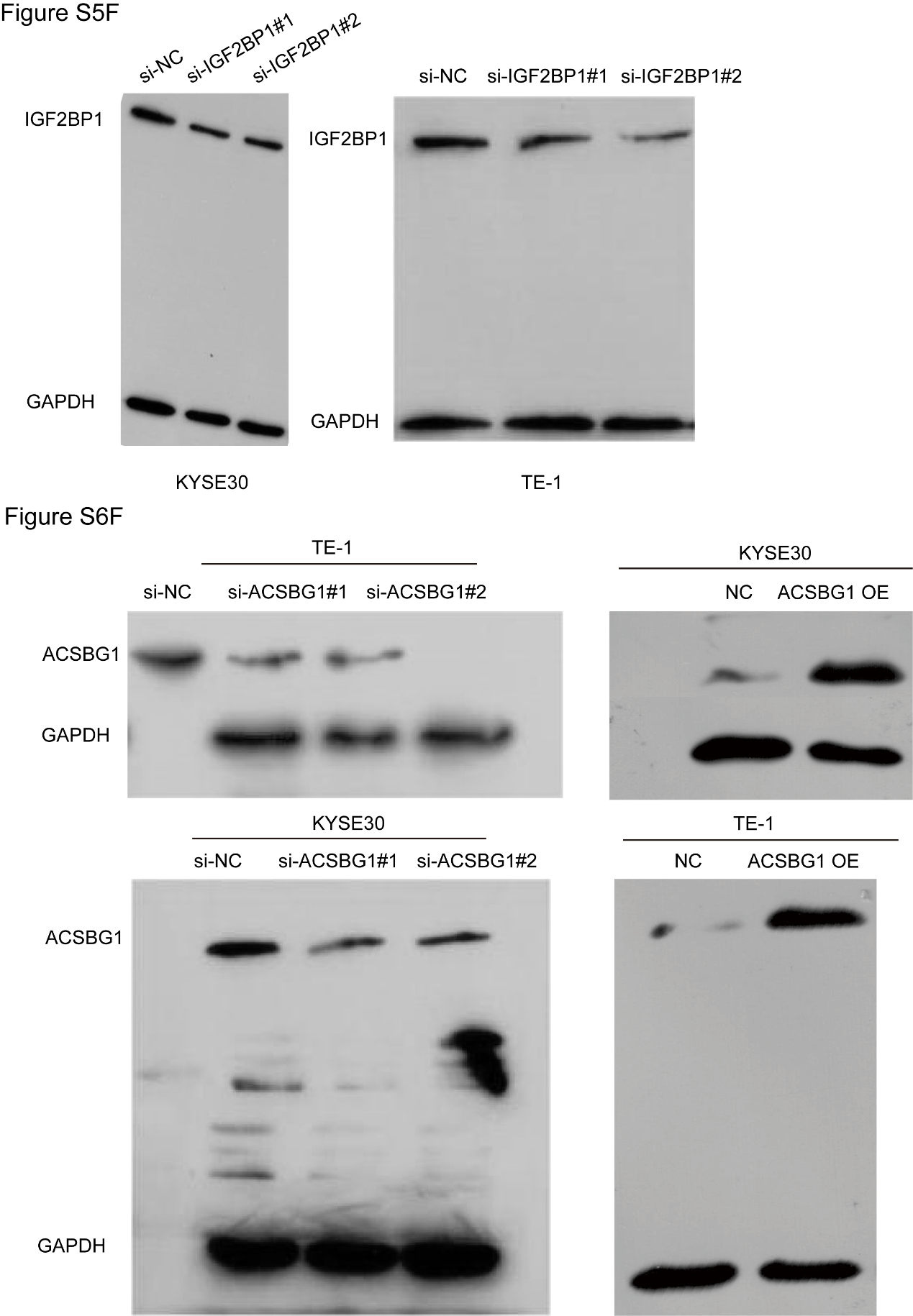


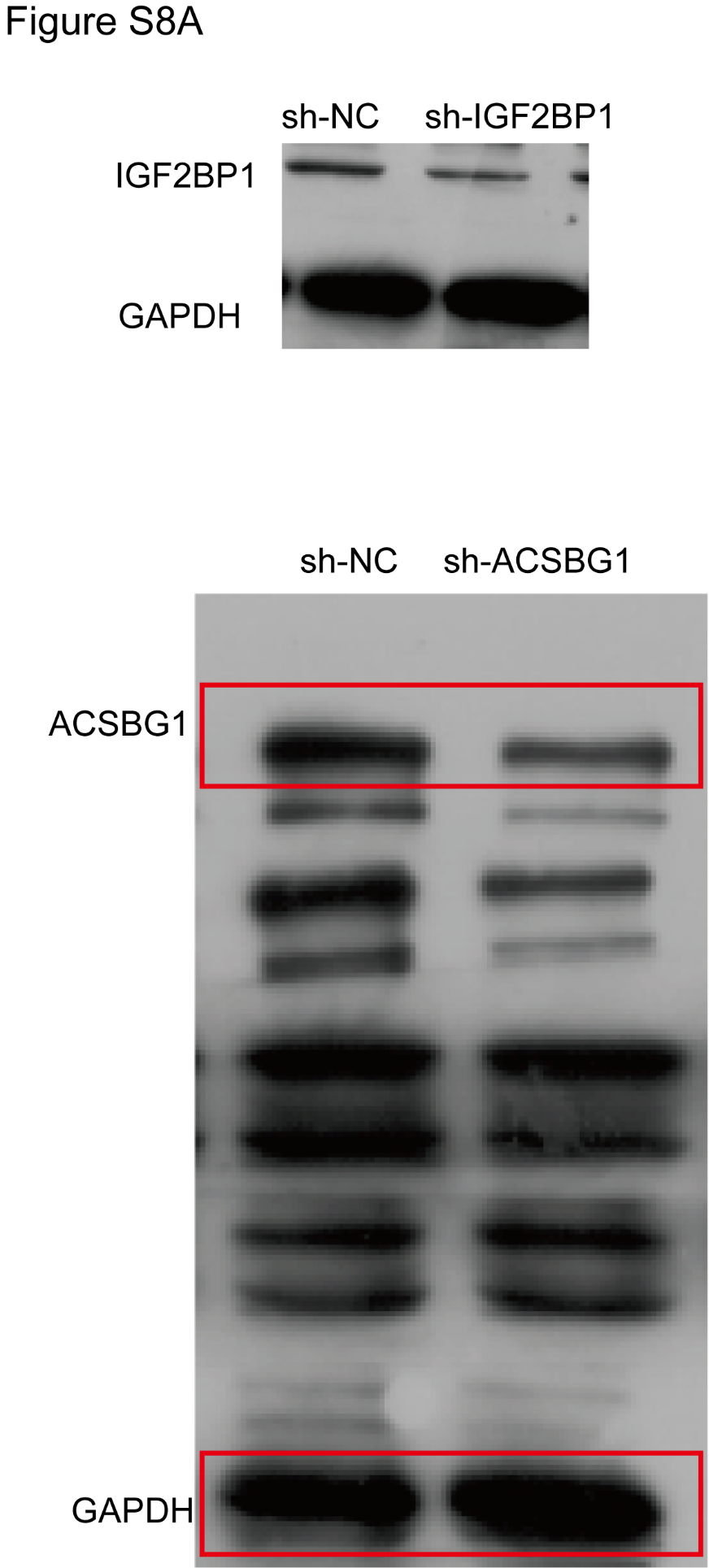

Supplement: Supplementary file 2 — Supplementary Material 2 [file 12885_2025_14390_MOESM2_ESM.docx]
