# Supplementary material for: CircSLC22A3 inhibits the invasion and metastasis of ESCC via the miR-19b-3p/TRAK2 axis and by reducing the stability of m6A-modified ACSBG1 mRNA
Source: BMC Cancer. 2025 May 30;25:971. doi: 10.1186/s12885-025-14390-8 (PMC12125856; doi:10.1186/s12885-025-14390-8)
Supplement: Supplementary file 3 — Supplementary Material 3 [file 12885_2025_14390_MOESM3_ESM.docx]

**Additional Figure
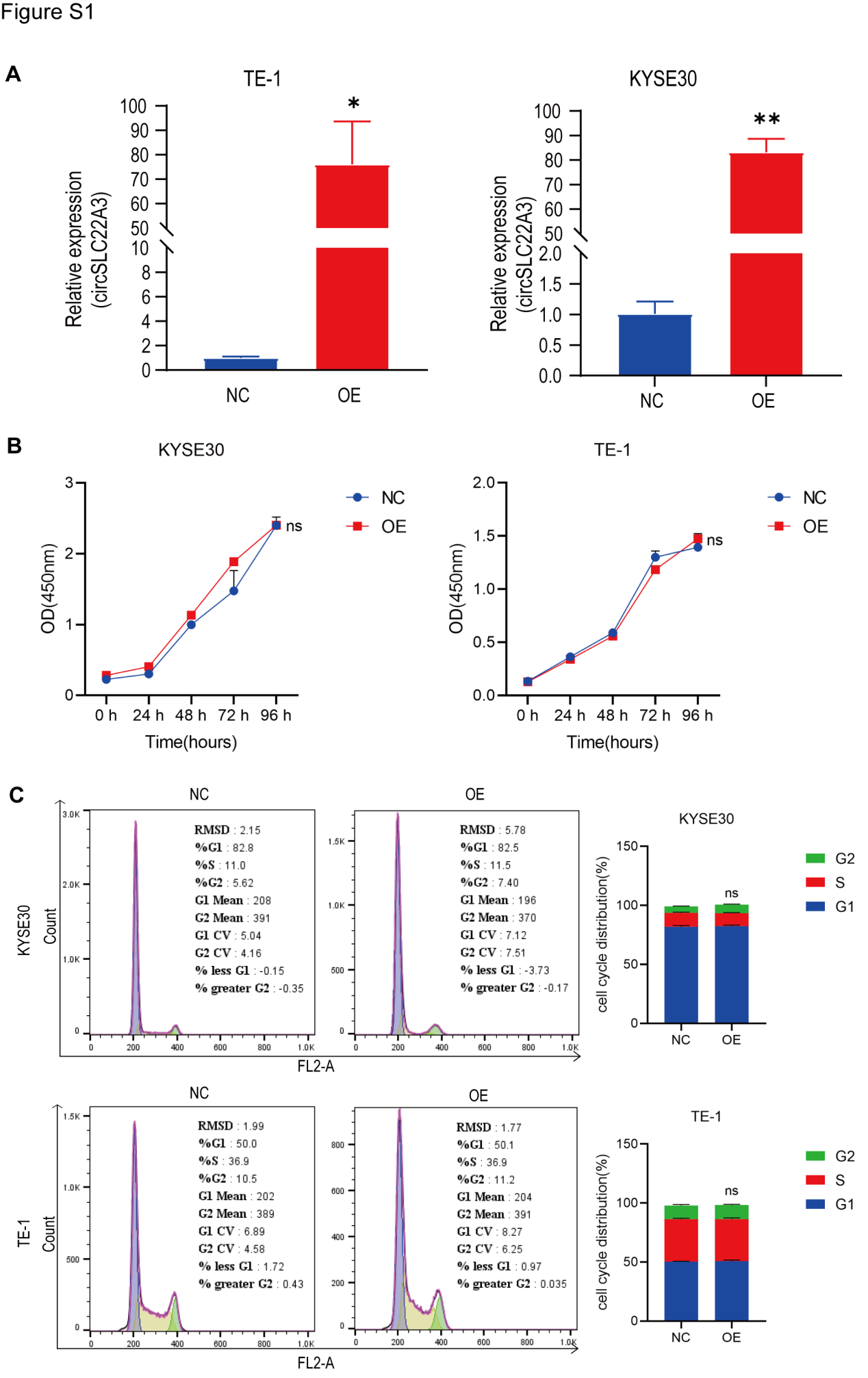
**

Figure S1 The overexpression of circSLC22A3 did not exert any influence on the proliferative capacity of ESCC

(A) CircSLC22A3 was successfully expressed in TE-1 and KYSE30 cell lines. (B) The impact of circSLC22A3 overexpression on the proliferative capacity of ESCC was assessed using a CCK-8 assay. (C) The influence of circSLC22A3 overexpression on the cell cycle in ESCC cells was detected by Flow cytometry analysis. *, *P* < 0.05; **, *P* < 0.01


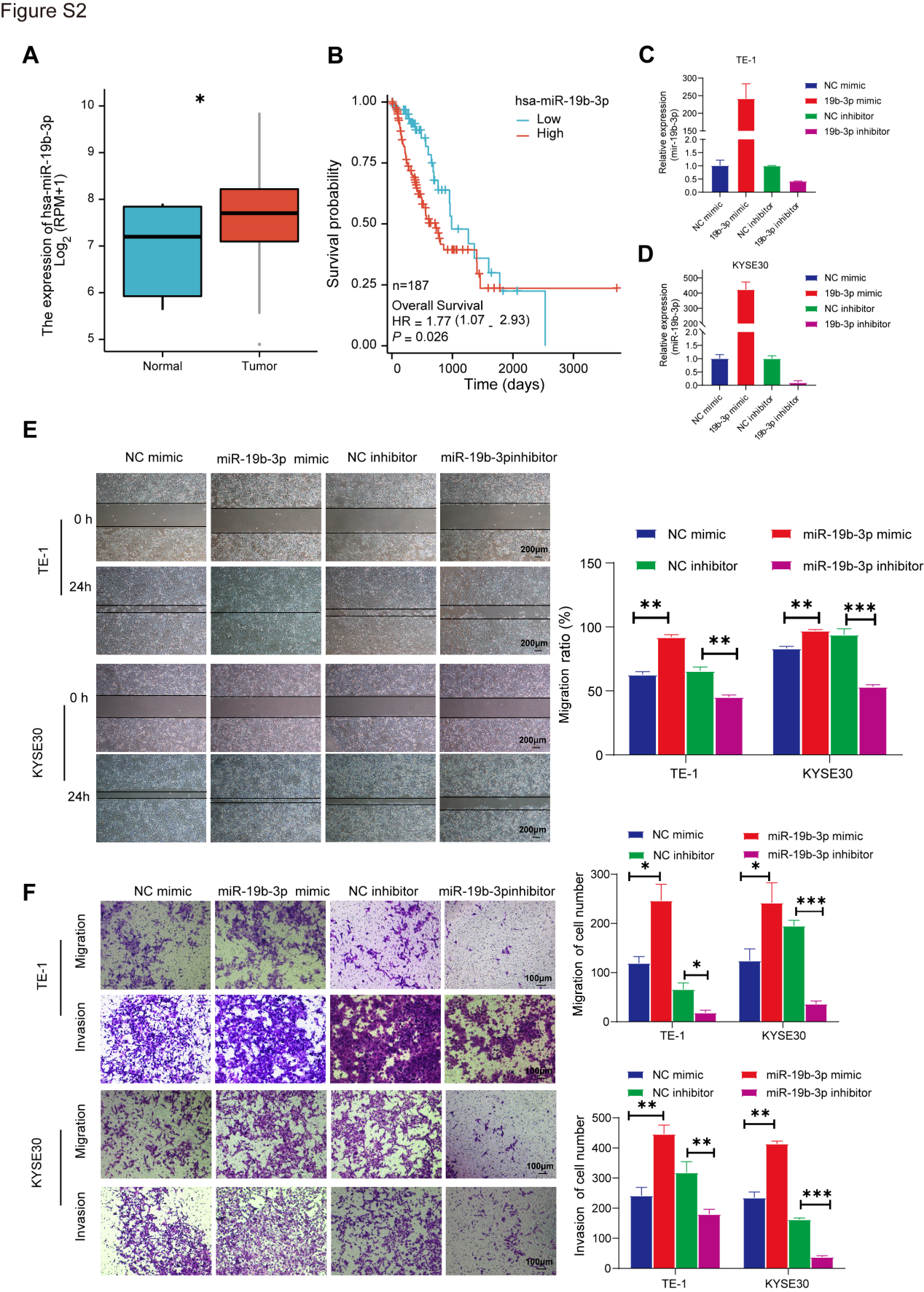


Figure S2 miR-19b-3p promoted the invasion and migration of ESCC cells

(A, B) The TCGA database showed that miR-19b-3p was highly expressed in ESCC, and patients with high expression of miR-19b-3p had worse prognosis than those with low expression. (C, D) The transfection efficiency was verified by qPCR. (E, F) miR-19b-3p promoted cell migration and invasion of ESCC. *, *P* < 0.05; **, *P* < 0.01; ***, *P* < 0.001.

**
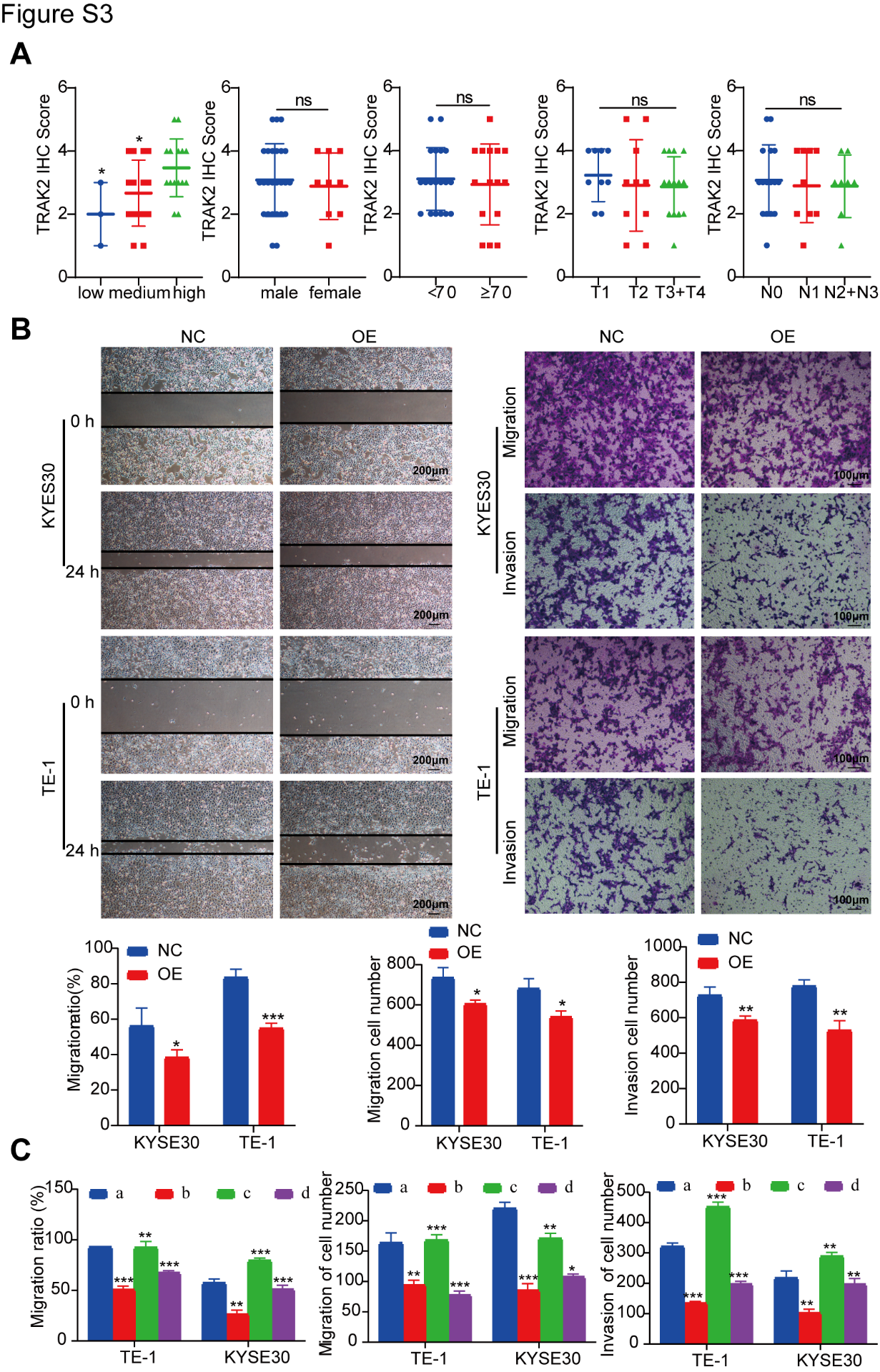
**

Figure S3 Overexpression of *TRAK2* inhibited invasion and migration of ESCC cells

(A) Analysis of the correlation between TRAK2 and clinical information of ESCC patients. (B) Inhibition of ESCC migration and invasion after overexpression of *TRAK2*. (C) Statistical analysis of cell scratch and Transwell rescue experiments on circSLC22A3, miR-19b-3p, *TRAK2*, group a is NC control, group b is circSLC22A3 OE, group c is circSLC22A3 OE+miR-19b-3p mimic, and group d is circSLC22A3 OE+miR-19b-3p mimic+TRAK2 OE. *, *P* < 0.05; **, *P* < 0.01; ***, *P* < 0.001.


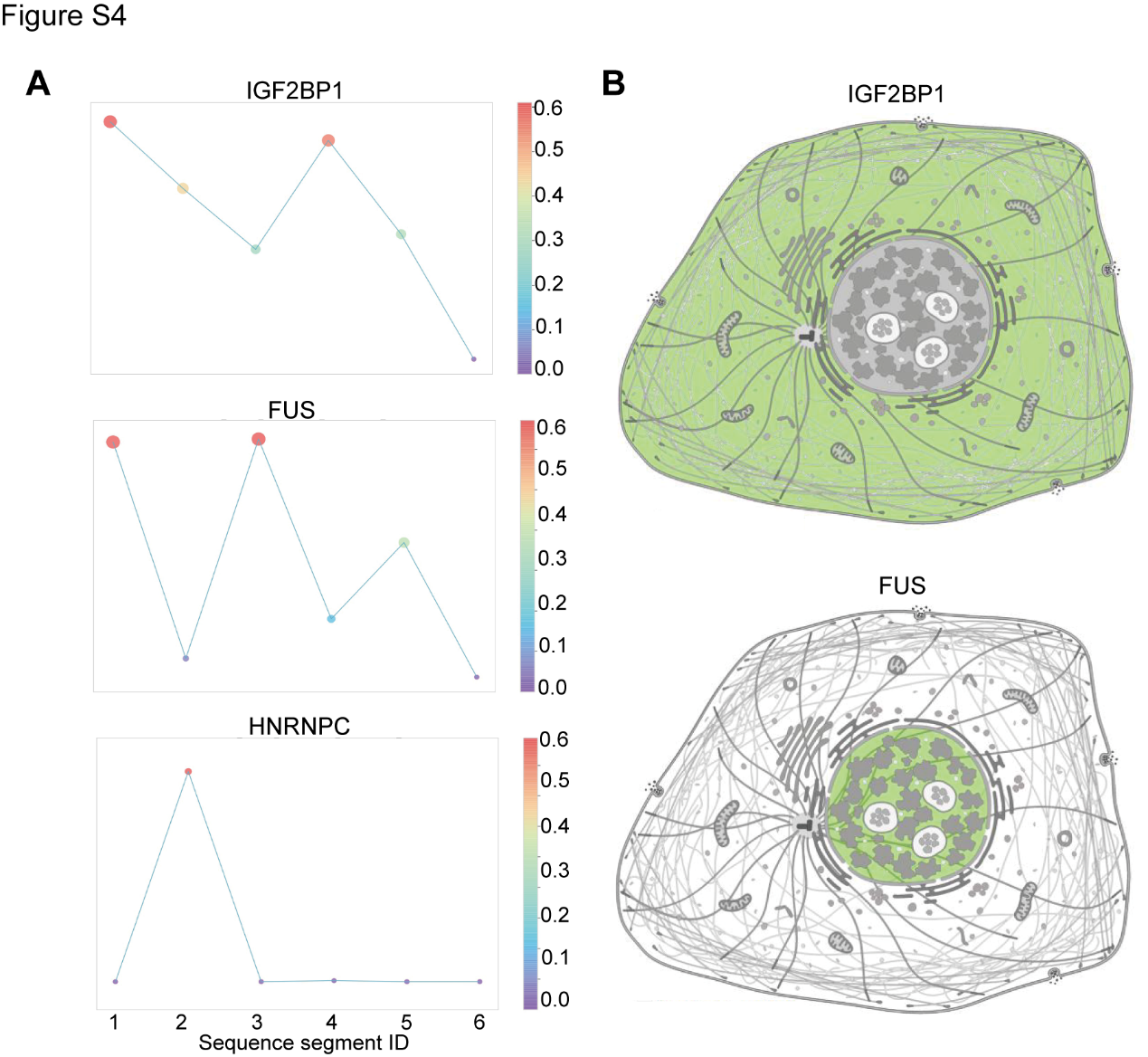


Figure S4 Predicted scores for the binding of circSLC22A3 with RBPs, and a schematic representation of the cellular localization of RBPs

(A) RBPsuite predicted the binding scores of circSLC22A3 with FUS, HNRNPC and IGF2BP1 respectively. (B) The Human Protein Atlas revealed that FUS and IGF2BP1 were localized in the cell nucleus.


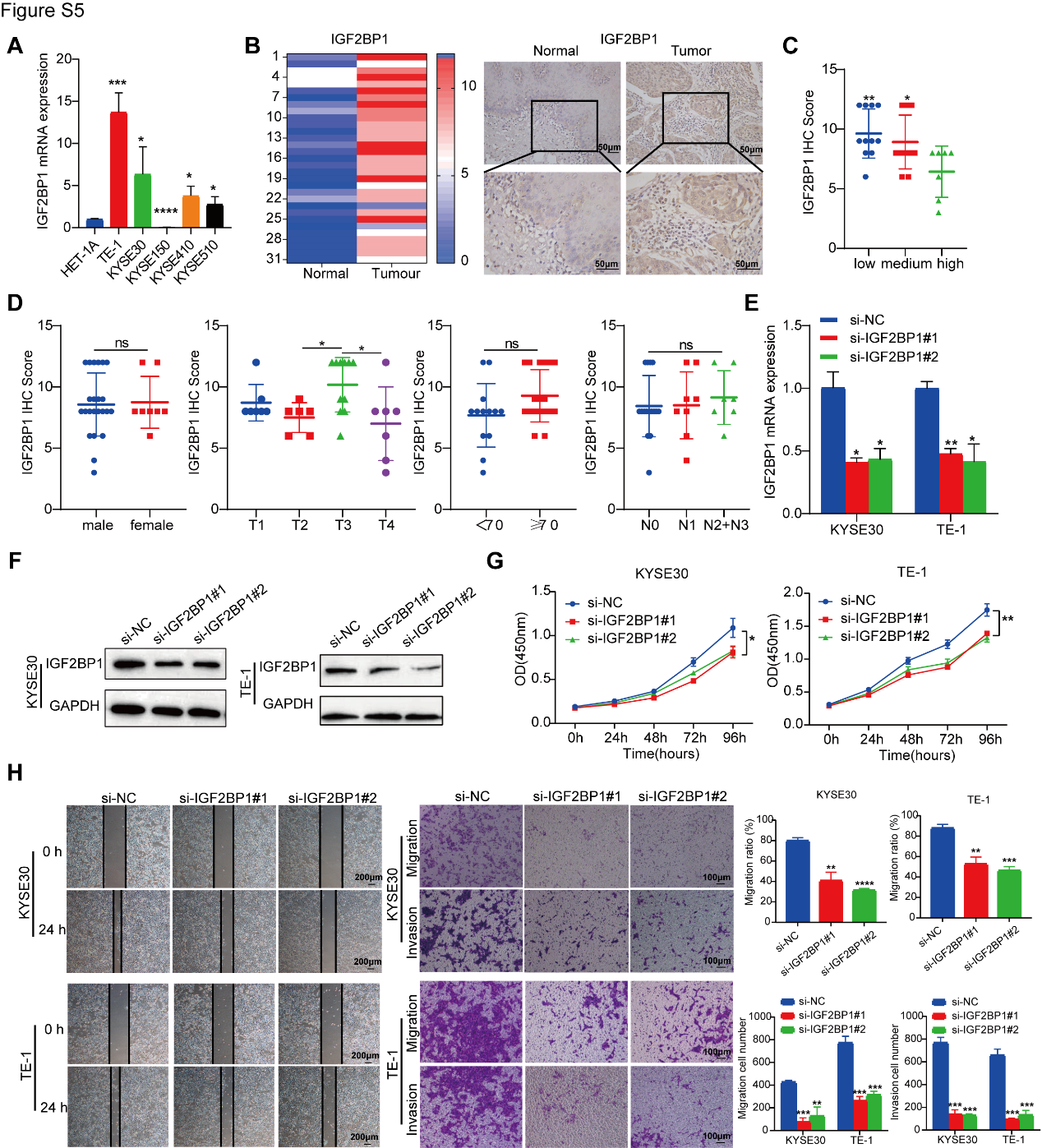


Figure S5 *IGF2BP1* is highly expressed in ESCC tissues and cells and promotes ESCC progression

(A) The expression of *IGF2BP1* was significantly increased in ESCC cells. (B) ESCC tumor tissues exhibited high expression of IGF2BP1. (C, D) Analysis of the correlation between IGF2BP1 and clinical information of ESCC patients. (E, F) IGF2BP1 was successfully downregulated at both the mRNA and protein levels. (G) Knockdown of *IGF2BP1* inhibited the proliferation of ESCC cells. (H) Functional assays demonstrated that IGF2BP1 promoted the migration and invasion of ESCC cells. *, *P* < 0.05; **, *P* < 0.01; ***, *P* < 0.001; ****, *P* < 0.0001.


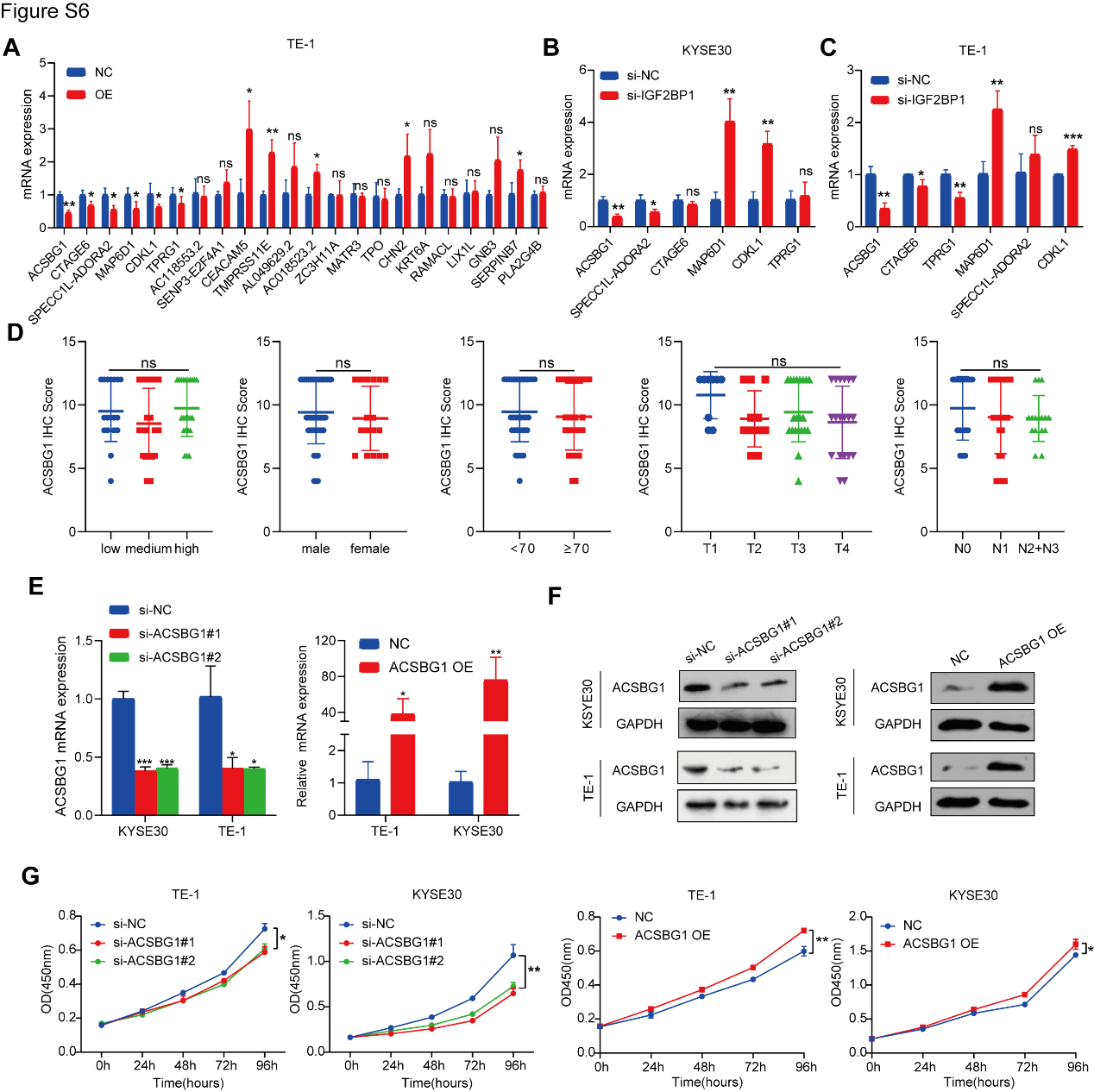


Figure S6 Screening of *ACSBG1* gene, clinical analysis and its promotion of ESCC proliferation

(A) RT-qPCR analysis revealed that the overexpression of circSLC22A3 in TE-1 cells resulted in a significant reduction in mRNA levels of six genes. (B) RT-qPCR analysis revealed that the knockdown of IGF2BP1 in KYSE30 cells resulted in a significant reduction in mRNA levels of two genes. (C) RT-qPCR analysis revealed that the knockdown of IGF2BP1 in TE-1 cells resulted in a significant reduction in mRNA levels of three genes. (D)Analysis of the correlation between ACSBG1 expression level and clinical information of ESCC patients. (E, F) ACSBG1 was successfully knocked down or overexpressed at both mRNA and protein levels. (G) Knockdown of ACSBG1 inhibited the proliferation of ESCC cells, and overexpression did the opposite. *, *P* < 0.05; **, *P* < 0.01; ***, *P* < 0.001; ****, *P* < 0.0001.


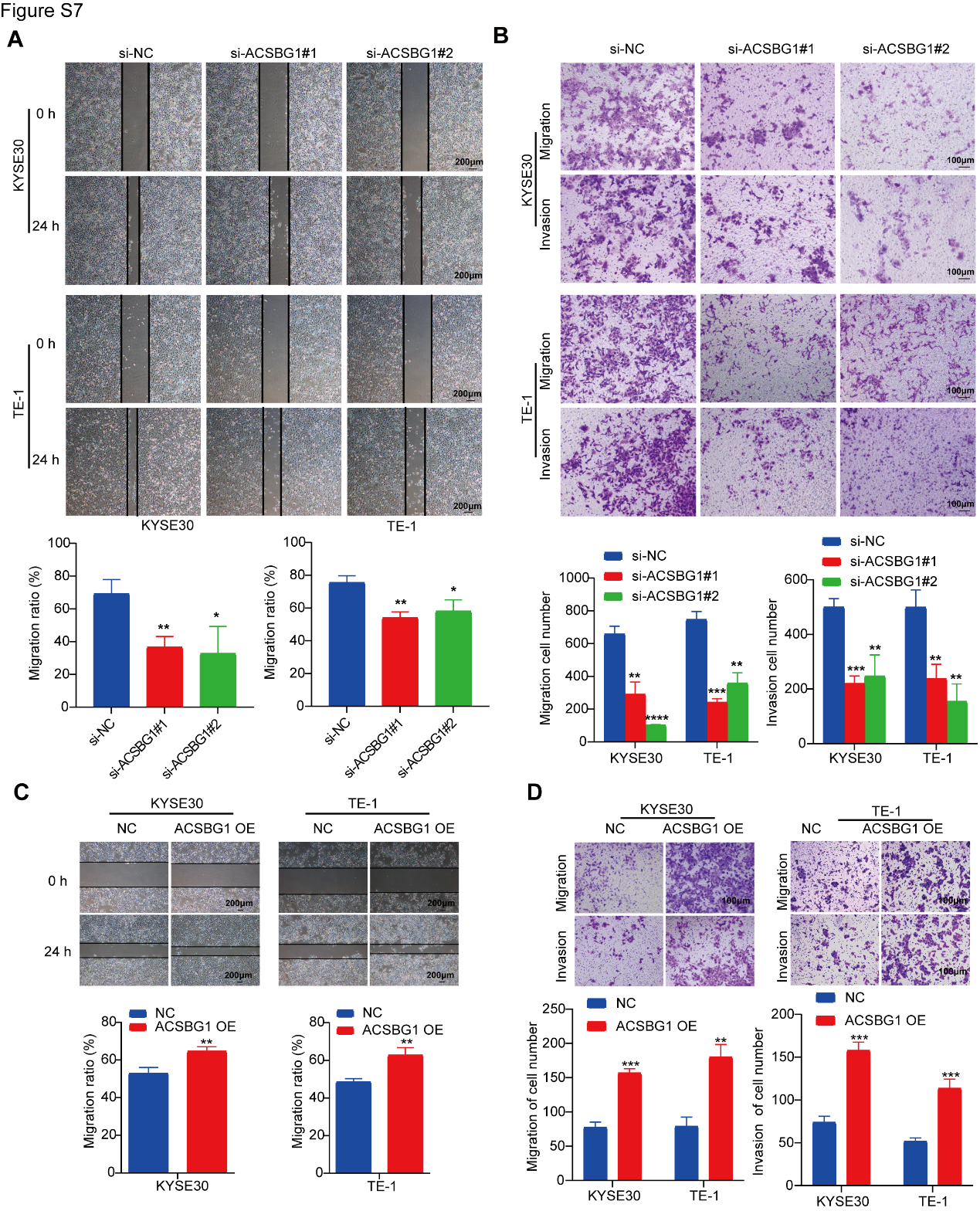


Figure S7 *ACSBG1* played a crucial role in facilitating the advancement of ESCC

*ACSBG1* plays a crucial role in promoting ESCC: (A, B) Knockdown of *ACSBG1* reduces ESCC cell migration and invasion. (C, D) Overexpression of *ACSBG1* increased migration and invasion of ESCC cells. *, P < 0.05. **, P < 0.01. ***, P < 0.001. ****, P < 0.0001.


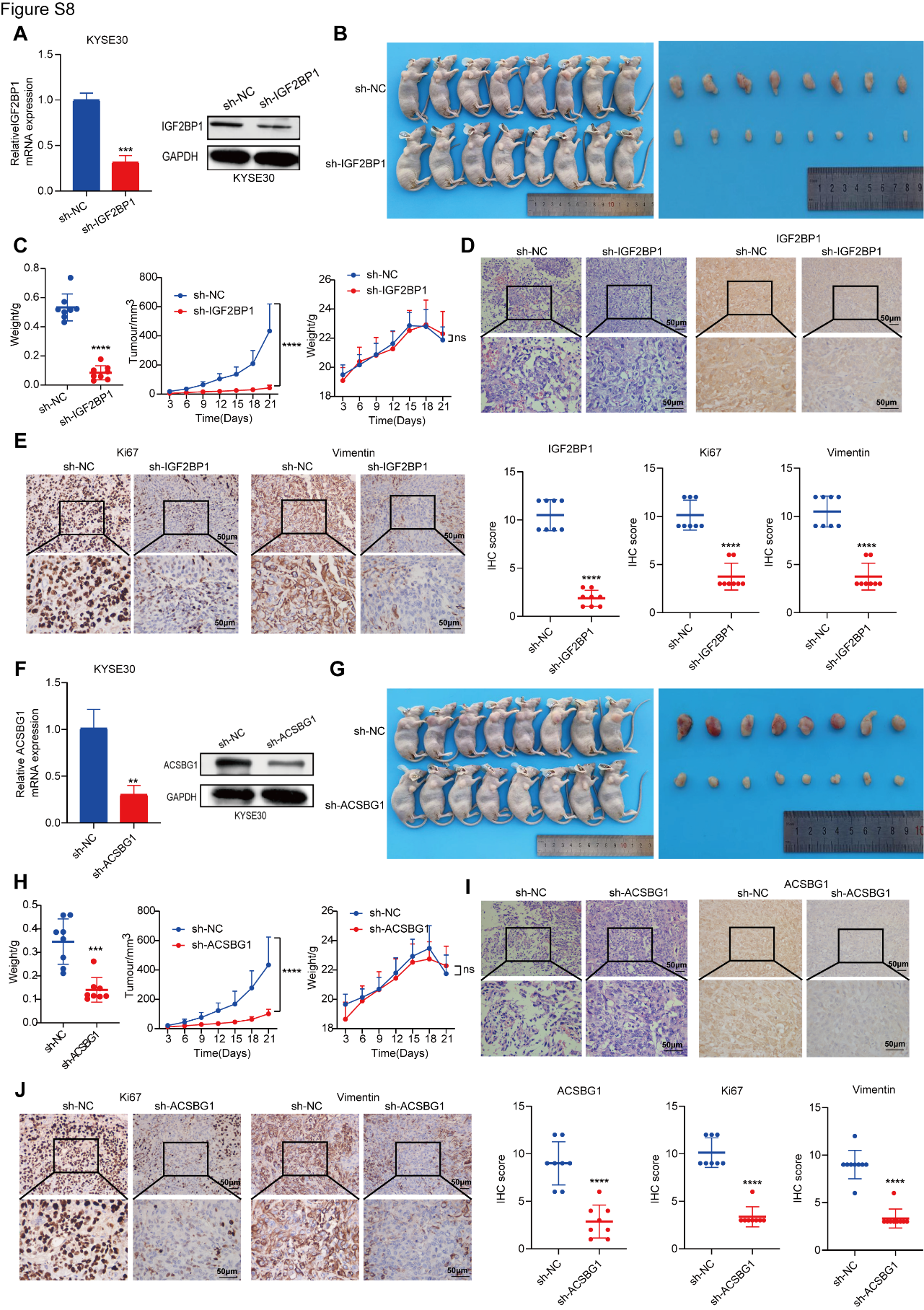


Figure S8 IGF2BP1 and ACSBG1 promote ESCC proliferation in vivo

(A) RT-qPCR and Western blot experiments were performed to validate the lentivirus-mediated knockdown efficiency of IGF2BP1. (B, C) In vivo, a decrease in tumor volume and body weight was observed in experimental groups knocking down IGF2BP1, but there was no significant effect on body weight in nude mice. (D, E) HE staining revealed that the tumor necrotic area and angiogenesis were reduced in the *IGF2BP1* knockout group, The *IGF2BP1* knockout group exhibited decreased protein expression levels of IGF2BP1, Vimentin, and Ki67. (F) RT-qPCR and Western blot experiments were performed to validate the lentivirus-mediated knockdown efficiency of ACSBG1. (G, H) In vivo, the group subjected to ACSBG1 knockdown exhibited a reduction in tumor size. (I, J) HE staining revealed that the tumor necrotic area and angiogenesis were reduced in the *ACSBG1* knockout group, The *ACSBG1* knockout group exhibited decreased protein expression levels of ACSBG1, Vimentin, and Ki67. *, *P* < 0.05; **, *P* < 0.01; ***, *P* < 0.001; ****, *P* < 0.0001.


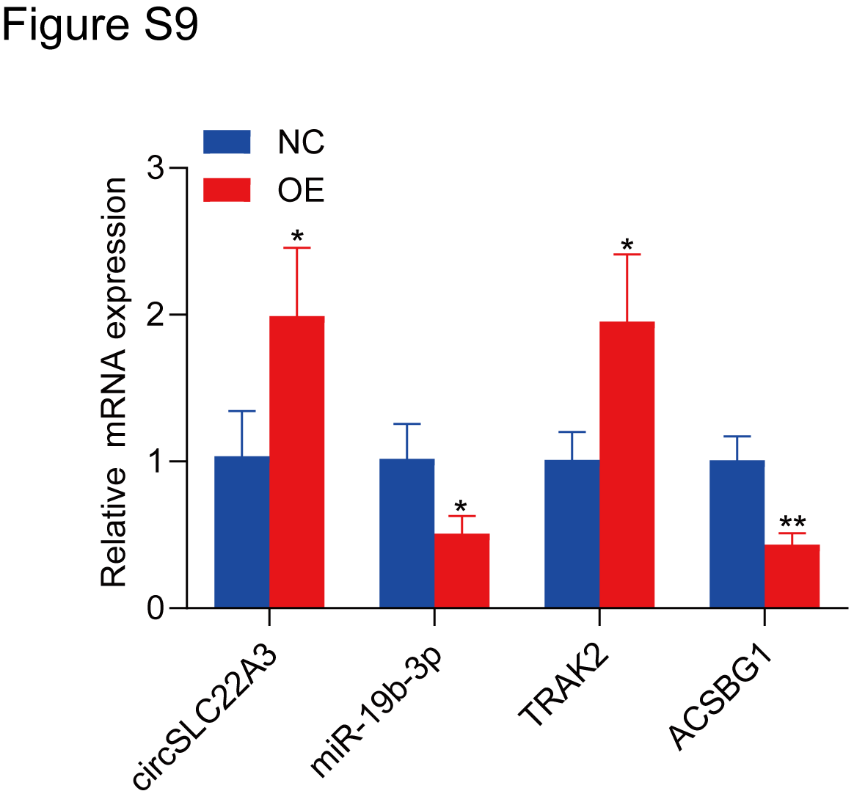


Figure S9 Correlation of circSLC22A3 with miR-19b-3p, TRAK2 and ACSBG1 expression in tissues of transfer mice

After overexpression of circSLC22A3, circSLC22A3 and TRAK2 expression levels increased, miR-19b-3p and ACSBG1 expression decreased, *, *P* < 0.05; **, *P* < 0.01.
